# Dietary Patterns of Children and Adolescents from High, Medium and Low Human Development Countries and Associated Socioeconomic Factors: A Systematic Review

**DOI:** 10.3390/nu10040436

**Published:** 2018-03-30

**Authors:** Patrícia de Fragas Hinnig, Jordanna Santos Monteiro, Maria Alice Altenburg de Assis, Renata Bertazzi Levy, Marco Aurélio Peres, Fernanda Machado Perazi, André Luís Porporatti, Graziela De Luca Canto

**Affiliations:** 1 Postgraduate Program in Nutrition, Health Sciences Center, Federal University of Santa Catarina, CCS/UFSC, Campus Trindade, Florianopolis 88040-900, Brazil; malicedeassis@gmail.com (M.A.A.d.A.); fernandamachado444@hotmail.com (F.M.P.); 2 Postgraduate Program in Human Nutrition, Faculty of Health Sciences, University of Brasília (UnB), Brasilia-DF 70910-900, Brazil; jordanna.santosmonteiro@gmail.com; 3 Preventive Medicine Department, University of São Paulo (FMUSP), São Paulo 01246-903, Brazil; rlevy@usp.br; 4 Australian Research Centre for Population Oral Health, The University of Adelaide, Adelaide, SA 5005, Australia; marco.peres@adelaide.edu.au; 5 Brazilian Centre for Evidence-Based Research Department of Dentistry, Federal University of Santa Catarina CCS/UFSC, Campus Trindade, Florianopolis 88040-900, Brazil; andreporporatti@yahoo.com.br (A.L.P.); delucacanto@gmail.com (G.D.L.C.)

**Keywords:** dietary patterns analysis, child, adolescent, socioeconomic factors, systematic review

## Abstract

The purpose of this systematic review is to assess the associations among education, income and dietary pattern (DP) in children and adolescents from high, medium and low human development countries (HHDC, MHDC and LHDC, respectively). Observational studies that evaluated the association between family income or education with the DP are obtained through electronic database searches. Forty articles are selected for review. In HHDC, education is inversely associated with “unhealthy” DP and positively associated with “healthy” DP. In cross-sectional studies from HHDC, higher income is negatively associated with “unhealthy” DP. In MHDC, there is no association between the socioeconomic variables (SE) and the DPs, although, in some studies, the unhealthy diet is positively associated with SE. Only one study conducted in LHDC showed an inverse association between income/education with “unhealthy” DP and there is no association between the SE and “healthy” DP. In conclusion, children and adolescents living in HHDC with high parental education tend to have a healthier diet. In MHDC, although an unhealthy diet is found among the high-income and educated population, the associations are not clear. Additional research is needed to clarify the associations between income and education with “unhealthy” and “healthy” DPs in MHDC and LHDC.

## 1. Introduction

Socioeconomic status (SES) has an influence on diet, regardless of age and the level of development of an individual’s country of residence [1,2,3,4,5]. In more economically developed countries, individuals with high-SES are more likely to consume healthy foods, whereas unhealthy diets are more commonly consumed by low-SES individuals [5,6,7].

As incomes rise and populations experience urbanization, societies enter different stages of the nutrition transition [8]. The nutrition transition refers to large changes in diet and activity patterns, especially in their structure and overall composition [9]. In relation to diet, there is a worldwide shift from traditional fiber and grain-rich diets to fat, sugar-rich, refined grains, animal fat and protein diets [8,10,11], leading to obesity and diet-related chronic diseases [12]. Many emerging and developing countries have also experienced this transition and its associated consequences on public health [13]. 

Associations between SES and diet in children and adolescents have typically been investigated via income or education [14,15]. The SES of the father, mother or head of the household is most often applied as a proxy for the assessment of the socioeconomic status of children and adolescents [16]. Income is one of the greatest indicators of material wealth [17]. Low income individuals may experience resource constraint and possible residential segregation in adverse food environments, leading to poor access to foods [18]. Education reflects material, intellectual, and other family resources [19] and may be linked to the acquisition, understanding, and implementation of knowledge about desirable dietary behaviors [18]. 

In recent years, the association between the SES and dietary pattern (DP) among children and adolescents has been studied [20,21,22,23,24,25,26]. Dietary patterns recognize that foods and drinks are consumed in combination, making it possible to study the whole diet. Most of these studies have been conducted in High Human Development Countries (HHDC); they have added to our understanding of the positive associations among education, income and a healthy DP in children and adolescents [21,24,26,27]. However, findings regarding the variables associated with an unhealthy diet have been contradictory [21,26,28,29] and Low and Medium Human Development Countries (LHDC and MHDC, respectively) [30,31,32,33] have been understudied. Evidence from a systematic review of studies with children living in LHDC and MHDC showed that there was a positive association between SES and obesity for both boys and girls, regardless of age, the level of gross national income (GNI) per capita, the level of obesity, the SES indicator chosen or the measure of fatness employed [34]. As DPs are important determinants of obesity [35], it is pertinent to explore the direction of the associations between DPs (healthy and unhealthy) and SES in children and adolescents from LHDC and MHDC. 

To our knowledge, no previous systematic review has examined the impact of a country’s socioeconomic development, using the Human Development Index (HDI), on the association between SES and DP among children and adolescents. Thus, we conducted a systematic review to answer two questions: (1) What are the known associations between SES and DP in HHDC? (2) Are there differences in the directions of the associations between SES and DP in MHDC and LHDC?

The first hypothesis of this systematic review is that education and income would be inversely associated with an “unhealthy” DP and positively associated with a “healthy” DP in children and adolescents living in HHDC. The second hypothesis is that education and income would be positively associated with unhealthy DPs in children and adolescents living in MHDC and LHDC. 

## 2. Materials and Methods

This systematic review was based on the standards outlined in PRISMA (Preferred Reporting Items for Systematic Reviews and Meta-Analyses) (Supplementary Material Table S1) [36]. 

### 2.1. Protocol and Registration

The protocol of this systematic review was registered at the International Prospective Register of Systematic Reviews (PROSPERO) under number CRD42015029562.

### 2.2. Eligibility 

#### 2.2.1. Inclusion Criteria

We included studies that evaluated the association between family income (household income or equivalent to family income) and/or education (maternal education, parental education or the highest level of education attained by any member of the household or the main food provider) and DP in children and adolescents (between 2 and 19 years old), with no language or time restrictions. 

Only observational (cohort, cross-sectional, case-control or ecological) studies that used statistical dimensionality reduction techniques to identify DP (cluster, factor analysis, principal components analysis, treelet transform, reduced rank regression, and latent class analysis) were included. All countries, regardless of the level of human development, were considered.

#### 2.2.2. Exclusion Criteria

The following exclusion criteria were applied: (1) studies that did not evaluate dietary patterns using statistical dimensionality reduction techniques and/or its association with family income and education; (2) studies of children under 2 years of age, adults or seniors; (3) reviews, letters, conference abstracts, case reports; and (4) intervention studies that may have affected DP.

### 2.3. Information Sources

Detailed individual search strategies were developed for each of the following databases: EMBASE (Excerpta Medica Database), Latin American and Caribbean Health Sciences (LILACS), PubMed, Science Direct, SCOPUS and Web of Science. A partial grey literature search was undertaken using Google Scholar and ProQuest Dissertations & Theses Global. The final search across all databases was updated on 22 January 2018. In addition, the reference lists of articles selected for full-text reading were hand screened for potentially relevant studies that could have been missed during the electronic database search. 

### 2.4. Search

Appropriate truncation and word combinations were selected and adapted for each database search (Table S2: Database search strategy). All references were managed by Endnote Web software version 3.1.1 (Basic-Thomson Reuters, New York, NY, USA) and duplicate hits were removed.

### 2.5. Study Selection 

Study selection was completed in 2 phases. In Phase 1, two reviewers Patrícia de Fragas Hinnig (P.d.F.H) and Jordanna Santos Monteiro (J.S.M.) independently reviewed the titles and abstracts of all references identified from databases. Articles that did not meet the eligibility criteria were discarded. In Phase 2, the same reviewers (P.D.F.H. and J.S.M.) applied the eligibility criteria to full-text articles. The lists of references from the selected studies were critically assessed by both examiners (P.D.F.H. and J.S.M.). Any disagreements were resolved by discussion until the two reviewers arrived at a mutual agreement. When they did not reach an agreement, a third examiner Maria Alice Altenburg de Assis (M.A.A.d.A.) made a final decision. The final selection was always based on the full-text of the publication.

### 2.6. Data Collection Process

Data were extracted independently by two reviewers (P.d.F.H. and J.S.M.). Again, any disagreement was resolved by discussion and mutual agreement among the two reviewers (P.d.F.H. and J.S.M.). To ensure consistency across reviewers, a calibration exercise was conducted before the beginning of the review. 

### 2.7. Data Items

The following information was recorded for each study, according to the levels of human development: country, survey year, follow up time points for cohort studies, study location, study design, the HDI that was recorded closest to the survey year, age range, sample size, dietary assessment method (DAM), dietary pattern method (DPM), SES indicator, DPs identified, and the direction of association between SES and DP. To test the hypotheses, we classified countries by predefined categories of HDI, low (LHDC: HDI ≤ 0.50), medium (MHDC: 0.799 ≥ HDI > 0.50), and high (HHDC: ≥0.80), according to the Human Development Report 2007/2008 [37]. We choose this reference year because 57.5% of selected studies were carried out between 2001 and 2010. Because of the HDI values used, three older studies, conducted in Portugal (2003–2004) [38] and England (1991–1995) [22,25] were classified as MHDC. 

The reported DPs vary according to country and the method used to define them. Three DPs were identified and used to describe the association between SES and DP: “unhealthy/Western”, “healthy/Mediterranean” and “traditional”. These patterns are most commonly used in the literature and are similar in terms of the types of food that compose each DP. Thus, in the present article we named the DPs according to the type of foods constituting each of the DPs. The DP composed of energy-dense, high-fat and low-fiber foods, snacks, fast foods, sweets, junk foods, treats, puddings, processed and ultraprocessed foods, the “Obesogenic” DP [31] and the “Healthy cluster at baseline, processed/sweet cluster at follow-up” DP [28] were labeled as “unhealthy/Western” DP. The DP with a higher intake of vegetables, fruits, whole grains, cereals, fish and olive oil, the “processed/sweet cluster at baseline and a healthy cluster at follow up” DP [28], the “guidelines adherence” DP [26], the “plant-based” DP [20], and the “meat and vegetable” DP [29], were labeled as “healthy/Mediterranean” DP. The “traditional” DP encompassed foods and preparations prevalent in the diet of the country where the study was conducted [39]. The “traditional” DP included dietary patterns labeled as “staple” DP [29], “varied Norwegian” DP [40], “local-based” DP [41], “family foods” DP [25] and “mixed diet” DP [30]. Some of the DPs identified in the revised studies were not classified and accounted for because they described mixed diets with different foods: “animal food intake” DP [42], “milk” DP [29], “beverages” DP [29], “dieting” DP [40], “combination” DP [43], “fish and sauce” DP [44], “dairy products” DP [45], “vegetarian” DP [46], “breakfast” DP [47], “monotonous” DP [47], “average fiber” DP [48], “starchy foods and drinks” DP [44], “vegetable soup, oil, butter, starchy foods, bread” DP [49] “lacto-vegetarian” DP [50], “transitive diet” DP [51], “egg and dairy” DP [52], “meat and chicken” DP [32], “fish, meat, processed meats, eggs, and starchy foods” DP [49], and “snack and fruit” DP [50]. Additionally, Danyliw et al. [53] identified beverage patterns that were not classified. 

The direction of the association between SES and DP was described and classified as follows: positive, inverse, none, U-shaped (intermediate exposure categories of a variable showing lower outcome frequencies than categories from higher and lower exposure), and not described. The positive, negative, and U-shaped associations were only described for those studies reporting statistically significant associations (i.e., if *p* < 0.05 or if the 95% confidence interval did not include a coefficient value of zero or an odds ratio value of one). We also described the results if no association was found.

To examine the differences in the directions of the associations based on the socioeconomic development of the country, firstly, we counted the number of times an association between income or education and DPs was tested for each HDI category. We then counted the number of times that this association was positive, negative or when no association was found. The results were also described and interpreted by the type of study design: cohort or cross-sectional. This approach was used because longitudinal examination of the data can provide further insights into changes in children’s and adolescent’s DPs and the identification of groups with persistently unhealthier diets that could be associated with SES [24,28].

### 2.8. Risk of Bias within Individual Studies 

The methodologies of the selected studies were evaluated using the Meta-Analysis of Statistics Assessment and Review Instrument (MAStARI) from the Joanna Briggs Institute for assessing risk of bias in comparable cohort, case-control and cross-sectional studies [54]. Two reviewers (P.D.F.H. and J.S.M.) independently assessed the risk of bias from each study. Disagreements between both reviewers were resolved by a third reviewer (M.A.A.d.A.). The risk of bias was categorized by the authors as “high” when the study reached a “yes” score up to 49%, “moderate” between 50% and 69%, and “low” when it was more than 70%.

In addition, the methodologies of dietary assessment (DA) were also analyzed because the method employed to obtain food consumption data may impact the dietary composition of each DP identified and consequently the direction of the associations between SES and DPs. At present, there is no universally accepted tool for scoring the methodological quality of DA. Each of the DA methodology presents its own strengths and limitations and there is no established method as a gold standard. Thus, to evaluate the quality of the dietary methodology used in each included study we first summarized in Table S3 the information provided by the authors regarding study population (age range and number of participants); DA method (type, recall/report period, structure of the tool in terms of number of food/beverages items and consumption frequencies categories, and the reporter of food/beverage consumption). We also searched and reviewed the validation studies cited in the primary papers (only for those that used the Food Frequency Questionnaire (FFQs) and added to Table S3 key characteristics of these studies (population and country where the validation study was conducted, reference method and results). Finally, we create a scale of scores from 0 to 10 based on literature recommendations to conduct an appropriate DA method in surveys with children and adolescents [55,56,57,58], considering the following attributes displayed in Table S3 related to: The type of DA method, the reporter of the diet, and the validation study for FFQs. A score was given for each study and a maximum score of ten points in each attribute reflected the better quality of the dietary assessment: Attribute 1—Dietary assessment method: (10) measurement of dietary intake using a method likely to represent usual dietary intake (i.e., a FFQ along with a 24-h dietary recall (24-h DR)/food diary (FD) or the 24-h DR/FD at least in 2 non-consecutive days; (5) studies that applied only the FFQ or one day 24-h DR/FD. Attribute 2—Validation study: (10) studies that the validation method of the FFQ was conducted in the same country and age group that the main study was performed; (5) the validation method of the FFQ was conducted in other country or age group; (0) the FFQ was not validated. Attribute 3—Reporter: (10) studies whose reporters were parents/caregivers (when children were ≤7 years old), parents/caregivers/plus child (when children were 7–10 years old) or only the adolescents or adolescent plus parents/caregivers (>10 years old); (5) studies whose reporters were only the parents/caregivers (when children were 7 to 10 years old or adolescents) or whose reporters were only the child (<10 years). The quality of assessment was categorized as “high risk of bias” when the study scored <20, “moderate risk of bias” when the study scored between 20 and 25, and “low risk of bias” when it was more than 25. Data from each study were extracted into a summary table and scored for methodological quality by two independent reviewers (P.d.F.H. and Fernanda Machado Perazi (F.M.P.), and disagreements were resolved by a third reviewer (M.A.A.d.A.).

## 3. Results

### 3.1. Study Selection

**Figure 1:**
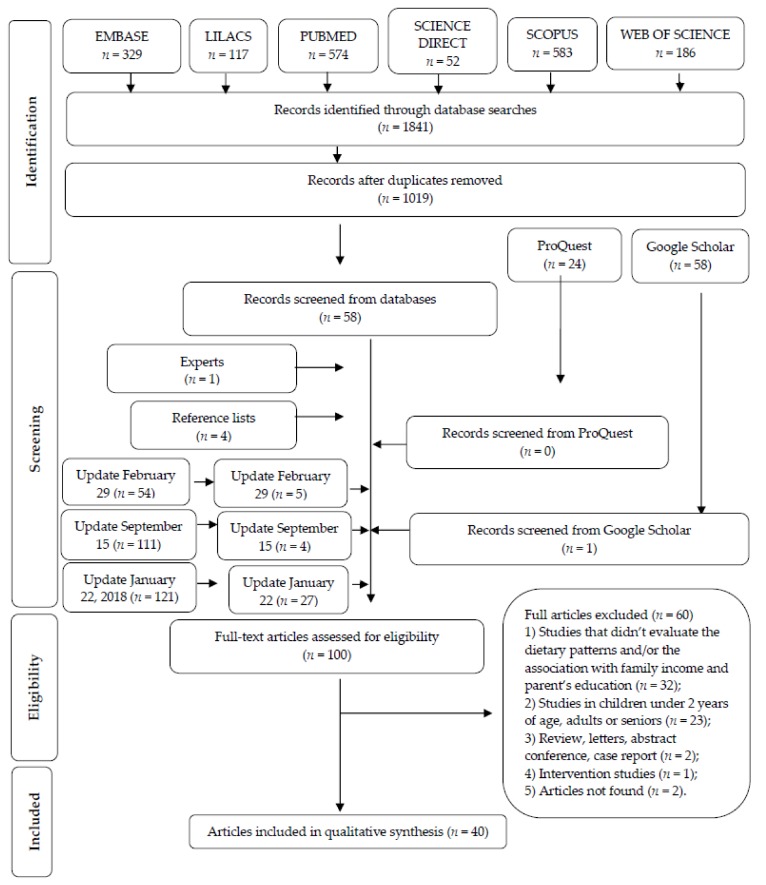
Flow diagram of literature search and selection criteria. Adapted from PRISMA [36].

In total, 1841 articles were initially identified across all electronic databases. After a comprehensive evaluation of the abstracts in Phase 1, a total of 58 articles were deemed potentially useful and selected for Phase 2 assessments. In addition, a total of 86 articles were identified from other sources: Google Scholar (*n* = 58), ProQuest (*n* = 24) and reference lists (*n* = 4). Of these 86 articles, 5 were deemed appropriate for Phase 2 assessment. An updated search conducted in February and September 2016, and January 2018 identified 286 new articles, 36 of which met the inclusion criteria in Phase 1. Five articles required a third examiner [28,48,53,59,60]. Experts suggested one additional articles [28]. Of the 100 studies selected in Phase 1, a total of 60 were subsequently excluded. Forty articles were retained for systematic review. Two studies identified for potential inclusion in this review (Bauce et al. [61] and Cairella et al. [62]) could not be evaluated as efforts to access the full-text articles were unsuccessful. A flow chart of the identification, inclusion and exclusion process is shown in Figure 1.

### 3.2. Study Characteristics

Table 1 presents a summary of seven cohort studies from HHDC and MHDC [21,24,26,28,29,42,63]. Table 2 presents a summary of 19 cross-sectional or cohort studies with cross-sectional analysis from HHDC [20,23,27,40,43,44,45,46,47,48,49,53,64,65,66,67,68,69,70], and Table 3 presents a summary of 16 cross-sectional studies from MHDC [22,25,30,31,32,33,38,41,47,50,51,52,60,70,71,72]. The studies conducted by Borges et al. [47] and by Manyanga et al. [70] included HHDC, MHDC and LHDC. No case-control studies were selected as none fit the eligibility criteria.

Twenty-five out of the 40 included articles were classified as HHDC [20,21,23,24,26,27,28,40,42,43,44,45,46,47,48,49,53,63,64,65,66,67,68,69,70] and seventeen as MHDC [22,25,29,30,31,32,33,38,41,47,50,51,52,60,70,71,72]. One Low Human Development Country (LHDC) was included by the study of Manyanga et al. [70].

Thirty-three articles had a cross-sectional design [20,22,23,25,27,30,31,32,33,38,40,41,43,44,45,46,47,48,49,50,51,52,53,60,64,65,66,67,68,69,70,71,72], and seven of the remaining articles were cohorts studies [21,24,26,28,29,42,63]. Sample size ranged widely, from 232 [30] to 18,046 subjects [60]. Twenty-three studies were carried out between 2001 and 2010 [23,26,27,28,29,30,31,38,40,42,43,44,45,46,47,49,50,51,53,60,63,68,69], eight were conducted before 2000 [20,21,22,24,25,64,65,67] and nine after 2010 [32,33,42,48,52,66,70,71,72]. The study conducted by Abdulla et al. [41] did not describe the year in which it was conducted.

Twenty-one studies included only children (2–10 years old) [20,22,25,26,29,30,40,42,43,49,50,52,60,63,65,66,67,68,69,71,72], ten included only adolescents (10–19 years old) [23,27,32,33,38,41,45,46,47,48] and nine included both children and adolescents [21,24,28,31,44,51,53,64,70]. All studies included both boys and girls; however, in six articles, the number of boys and girls was not specified [24,29,30,66,67,68]. Parental education was used as the exposure variable in 36 articles [20,21,22,24,25,26,27,28,29,30,31,32,33,38,40,41,43,45,46,47,48,49,50,51,52,60,63,64,65,66,67,68,69,70,71,72], one article used education of the main food provider [44] and another used household education [53]; income data were provided by 18 articles [23,26,27,28,30,31,32,33,41,42,44,47,51,53,60,69,70,71].

Principal components analysis (PCA) was the method most frequently used to identify the DPs (*n* = 27/40) [22,23,25,26,29,30,31,32,33,40,41,42,43,44,45,46,47,49,50,52,60,63,64,65,66,67,70], followed by cluster analysis (CA) (*n* = 8/32) [20,24,28,38,48,51,53,68], reduced rank regression (RRR) (*n* = 1/32) [21], factor analysis (FA) (*n* = 3/32) [27,51,71], and latent class analysis (LCA) (*n* = 1/32) [69].

The “unhealthy/Western dietary pattern” was identified in 37 articles [20,21,22,23,24,25,26,27,28,29,30,31,32,33,38,40,41,42,44,45,46,47,48,49,51,52,60,63,64,65,66,67,68,69,70,71,72], followed by the “healthy/Mediterranean dietary pattern” (*n* = 30/40), and the “traditional” DP (*n* = 15/40) [20,22,25,29,30,31,33,40,41,46,47,65,67,71,72].

### 3.3. Risk of Bias within Individual Studies

One article fulfilled all of the methodological quality criteria [46]. Ten articles were classified as having a low risk of bias [20,21,25,33,46,47,52,66,68,72], 22 articles had a moderate risk [22,24,26,27,28,29,30,38,41,42,43,44,45,49,50,51,53,60,63,65,69,71], and eight articles presented a high risk [23,31,32,40,48,64,67,70]. More information about the risk of bias is shown in Table S4: Risk of bias was assessed by Meta-Analysis of Statistics Assessment and Review Instrument (MAStARI) critical appraisal tools. 

Table S3 summarizes the key characteristics of studies included in the review in relation to the dietary methodology used, which supported the assessment of the quality of how the food data were collected. Three articles used a FFQ along with a 24-h DR to collect the food consumption [23,45,64], 27 (67.5%) articles used only a FFQ [20,22,25,26,27,28,29,30,31,32,33,38,40,41,42,44,46,48,49,50,60,63,66,67,69,70,71], five articles used only a 24-h DR [43,47,51,53,72] and the remaining five articles used FD [21,24,47,52,68]. The parents or caregivers were the most used reporters in studies conducted with children until seven years old [20,21,22,24,25,26,28,29,30,42,49,60,63,65,66,67,68,69,71,72]. In studies conducted with children in the age range 7–10 years, eight studies used the parents or caregivers plus the child as the reporter [21,24,43,44,50,52,53,64], and in six studies the child was the single reporter [20,28,40,49,65,67,71]. Most studies conducted with adolescents used only the adolescents as a reporter [23,31,32,33,41,45,47,48,51,53,64,70]. While some studies have used validated dietary intake assessment tools whose studies were conducted in the same country and age-group as the main study [27,28,31,33,41,44,64,66,69,70], others reported a validation method performed in a different country or age group [20,22,23,25,26,38,42,45,46,48,49,63,67], and a number of others did not report the use of such tools [29,30,32,40,50,60,71]. The majority of the included articles (*n* = 24, 60%) [20,22,23,25,26,27,28,31,33,38,41,44,45,46,48,51,53,63,65,66,67,69,70,72] were classified as “moderate risk of bias”, followed by a “high risk of bias (*n* = 9, 22.5%) [29,30,32,40,42,49,50,60,71]. Only seven studies were classified as “low risk of bias” indicating the better quality of the dietary assessment in these studies [21,24,43,47,52,64,68].

### 3.4. Synthesis of Results

#### 3.4.1. Cohort Studies from High and Medium Human Development Countries (HHDC and MHDC, Respectively)

In cohort studies from HHDC, an inverse association between education and “unhealthy” DP was reported 7 out of 11 times; for the other four times no association was found. A positive association between education and “healthy” DP was reported for seven out of eight times. An inverse association was recorded between income and “unhealthy” DP half of the time (two out of four), and no associations between income and “healthy” DP was found in three out of four times (Table 4). One cohort study from a MHDC (Brazil) [29] found an inverse association between education (primarily maternal education) and unhealthy DP (“snack” and “treats” DPs) and traditional DP (“staple” DP), and a positive association between education and the healthy DP (“meat and vegetables” DP) (Table 1).

#### 3.4.2. Cross-Sectional Studies from High Human Development Countries (HHDC) 

Education and/or income and “healthy” DP were positively associated 12 out of 22 times, and eight out of 14 times, respectively. Inverse associations were reported between education and “unhealthy” DP 22 out of 32 times, and between income and “unhealthy” DP 10 out of 15 times. In five out of eight studies, no association between education and “traditional” DP was found. No cross-sectional study from HHDC assessed associations between income and “traditional” DP (Table 4).

Danyliw et al. [53] identified beverage patterns among Canadian children aged 2–18 years old and compared these patterns with sociodemographic characteristics. For boys aged 6–11 years old in low household income families, the prevalence of the “high-fat milk” DP was highest compared to the other beverage DPs (“Soft drink” = 14.9%, “Fruit drink” = 9.6%, “Fruit juice” = 7.3%, “Milk” = 7.1%, “High-fat milk” = 22.0%, “Moderate-fat milk” = 13.8%, *p* = 0.037).

#### 3.4.3. Cross-Sectional Studies from Medium and Low Human Development Countries (MHDC and LHDC)

A positive association between income and “unhealthy” DP (five out of 19 times) and between education and “healthy” DP (seven out of 17 times) was observed. No association between education and “unhealthy” DP was found 11 out of 27 times, eight times this association was positive (29.6%) and in the same number of studies (eight out of 27) this association was inverse. Mainly, there was no association between income and “healthy” DP. The relationship between education or income and “traditional” DP was also studied in MHDC, but most articles did not report any association. In India, the authors described no association between the “mixed” DP (“snack and fruit” and “lacto-vegetarian”) and education [50]. The only study conducted in LHDC (Kenya) showed an inverse association between income and education with “unhealthy” DP and no association between SES variables and “healthy” DP [70].

## 4. Discussion

Three main results can be drawn from this systematic review: (1) In accordance with our first hypothesis, cohort and cross-sectional studies conducted in HHDC found that education was inversely associated with the “unhealthy” DP and positively associated with the “healthy” DP. (2) Cross-sectional studies conducted in HHDC found that higher income was associated with lower adherence to “unhealthy” DP. (3) Contrary to our second hypothesis, the majority of cross-sectional studies conducted in MHDC found no association neither between education or income and “unhealthy” DPs, nor between education or income and “healthy DP”. In some studies, the unhealthy diet was found to be positively associated with the high-income/educated population and also with the low-income/educated population.

The rise in rates of obesity and other non-communicable diseases (NCD) are highest in countries and regions undergoing rapid socio-economic changes (e.g., India, Brazil, China, Middle East, North Africa, and Southern Africa). Such patterns are also evident in children and adolescents from MHDC and LHDC [73,74,75,76,77]. Therefore, the specific novelty of this study is that unhealthy DP, considered to be major risk factor for NCD development [78] was identified in 92.5% of the studies included in this systematic review. In developing countries, such a pattern was identified in almost all studies conducted with children and adolescents, and 26.3% of the time, the unhealthy DP was positively associated with income.

In a systematic review conducted by Mayén et al. [5] to assess the social dietary patterns of adults from low-middle income countries, the authors found higher fat consumption and lower fiber intake in adults with high SES. The authors hypothesized that a fat-rich diet was associated with increased prosperity [8] and supermarket expansion [9,79]. As well, changes in consumption patterns, as a result of individuals working outside of their homes, was associated with fiber-poor diets, which also likely reflects on children’s and adolescents’ diet [80]. In our review, most of the MHDC studies were conducted in Brazil, where eating outside of the home has gained importance; ultra-processed foods, energy, saturated fat, trans fat, carbohydrates and free sugar intake are higher in food eaten out of home, whereas fiber and iron intake are reduced [81,82]. In the study conducted by Andrade et al. [82] in Brazil, it was found that adherence to the “ultra-processed food” DP rises according to the increase in education and income. 

Income and food price are the two most influential factors leading to dietary convergence in developing countries [83]. While globalization is an opportunity for a higher intake of healthy and varied foods in transitioning economies, it also allows for greater consumption of low-priced, energy-dense items [5]. The access to surplus/excess food lead to an increased consumption of saturated and trans fats, sugars, salt and processed foods that contain excessive amounts of these components [83,84]. However, the social pattern of a diet may reverse with the progression of nutrition transition. Indeed, along with socioeconomic development, people with a low SES tend to adopt unhealthier diets, as suggested by data on obesity prevalence and its association with a higher consumption of energy-dense foods (e.g., soft drinks and ultra-processed food) [1]. 

In the present study, the observed positive association between education and the “healthy” DP in HHDC was also described in a review by Smithers et al. [85] conducted with toddler and preschool aged children. According to Dinsa et al. [34], highly educated individuals in these countries are more likely to be health-conscious and able to afford and maintain a healthier diet. A meta-analysis showed that fruit and vegetable consumption was consistently higher in the high than the low SES group [86]. Education might influence food choice by facilitating or constraining one’s ability to understand and interpret health-related information communicated through nutrition education messages or on food labels [87]. Maternal education may impact child survival, including the mother’s ability to: contribute to the family’s income; command authority and make decisions in the family; make use of existing services; and provide child care [3]. In this systematic review, the association between income and “unhealthy” DP was not clear, whereas cross-sectional studies found that the “unhealthy” DP was inversely associated with income. The inverse association may be a result of the high cost of healthy diets [78,88,89].

This review suggests that, as countries grow economically, there is a greater adherence to an unhealthy diet in children and adolescents of less educated parents. Lack of food becomes less common even amongst society’s poorer strata after a certain stage of economic growth has been reached. The lower educational level and limited health-related knowledge of the poor compared to those with a high SES is coupled with a greater difficulty in acquiring more expensive and less energy-dense foods [90,91]. 

We found that using education and/or income as a SES indicator was relevant in assessing the association between SES and the DPs. The direction of this association was most evident for education in HHDC. Monsivais and Drewnowski [92] found that education was a stronger predictor of the consumption of energy-dense foods than household income in American adults. Maternal educational is a significant predictor of healthy DPs, because mothers generally spend significant time interacting with their children [93]. Moreover, education as an indicator of SES is more stable over time than income [94]. For tax or security reasons, or even due to embarrassment, respondents may be reluctant to report their real earnings [15]. In addition, not all financial resources are earned through income, usually understood as direct labor compensation [95].

The classification of countries by HDI is a more appropriate indicator of “development” than Gross National Income (GNI) per capita [34] because GNI cannot satisfy all aspects of development [96]. The HDI reflects both social and economic development and was created to emphasize that people and their capabilities should be the ultimate criteria for assessing the development of a country, rather than economic growth alone [37]. 

Regarding the period covered by this systematic review, we identified that only nine out of the 40 studies were carried out after 2010. Of these studies, six were conducted in MHDC. Some studies conducted up to the year 2010 showed a positive association between education or income and the “unhealthy” DP. After 2010, there was an increase in the number of studies that did not show any association between these variables, as well as a reduction in the number of studies that found a positive association between SES and “unhealthy” DP. These findings could indicate that in MHDC, the nutrition transition may be nearly complete [70]

The strengths of this review are the inclusion of studies from different countries and the large sample sizes used. These allowed us to assess the association between the socioeconomic variables and dietary patterns in a variety of countries, with different levels of economic development. It also assured diversity among participants (sex and age) and the representativeness of the findings. Furthermore, despite the different statistical methods used to identify the DP, similar results regarding the associations among education, income and DP were found. The DP methods combined information from the whole diet, considering the complexity of eating behavior, revealing underlying food consumption patterns and providing more relevant information about dietary choices than analyses based on the consumption of individual foods and/or nutrients [44].

The associations between the socioeconomic variables and the dietary patterns are questionable because most studies included in our systematic review are cross-sectional. Associations found in cross-sectional studies are not, by themselves, evidence of causality [78]. However, children with more educated parents could be over-represented in the cohort studies and this may limit generalizability of the results [63]. Moreover, education is more stable over time than income, which leads us to believe that parental education precedes the outcome. Another major finding appears to be the shortage of cohort studies undertaken in MHDC and LHDC, suggesting that more longitudinal studies are required, especially in developing countries.

Moreover, 75% of studies had a moderate to high risk of bias assessed by MAStARI and may have over or underestimated the true effect of the exposure variable [54]. Furthermore, the assessment of the quality of dietary methodology in the included studies showed that few studies used optimal strategies to enhance accuracy of the reported food intake. Validation of dietary methods was not conducted in several of the studies reviewed, which may have implications for the accuracy and reliability of the findings. Dietary assessment of children is challenged by the fact that they tend to have diets that are highly variable from day to day, and their food habits can change rapidly [55,57,58]. In school-age children, the cognitive abilities for self-reporting, good memory, spelling and reading competencies, attention required as well as the time concept required for a comprehensive dietary intake review are not yet fully developed, and they may need help from their parents to self-complete a dietary assessment [55,58,97]. Some of the studies in school age children used only the parents/caretakers or the child as the reporter. Furthermore, assessment of dietary intake of adolescents is influenced by underreporting and misreporting, which is common among overweight and obese adolescents [98]. 

The main limitations of the common methods of assessing dietary intake center on the accuracy of the data obtained by such methods in estimating an individual’s usual dietary intake [99]. Whereas a single 24-h recall or record is appropriate for estimating the average dietary intake of a population, at least two days of recalls/records are needed to model estimates of the population’s usual intake distributions and their relationships with other factors [99]. Because the foods consumed on consecutive days of reporting may be related, it is advisable to collect nonconsecutive single-day records or recalls to increase representativeness of the individual’s diet. Although the FFQ asks about the respondent’s usual intake of foods over an extended period, the estimation tasks required for a FFQ are complex and difficult, especially for children [97]. It has also been suggested that FFQ data might be combined with recall or record data to improve estimated intakes. Thus, blended instruments are now being recommended to enhance the quality of the dietary assessment intake in population surveys with children and adolescents [58]. The scores applied to analyze the quality of DA methodologies in the included studies of this review were based on only three attributes and in arbitrary decisions regarding cut-offs for scoring and the scaling of scores. We acknowledged that there are other attributes inherent to DA methodologies that may help to analyze the quality of DA methodologies.

This review synthesized the directions of the association between education, income and DP, not the strengths of these associations. We were unable to perform a formal meta-analysis because it was not clear whether the underlying data and methods were comparable enough to allow for quantitative analysis. The statistical techniques used to identify DP require that arbitrary decisions and the subjective interpretation of factors be made [27,44].

Understanding how DP may be associated with SES in countries with different levels of human development is important for informing policy makers on improving education systems and interventions to promote healthier diets. Our results from MHDCs suggest that interventions should be developed for children and adolescents of all SES, including those from high SES (and who study in private schools), due to the observed results showing that the unhealthy diet is still a problem in richer populations. 

Nutrition education should focus on food-based dietary guidelines (FBDG) instead of nutrients only. Such a strategy would facilitate the translation of ideas about a healthier dietary pattern to the public because FBDG are usually clearer and easier to understand compared with nutrient-based approaches, and this may be particularly true for young children and their caregivers [100,101]. Moreover, the messages should encourage parents and schools to take responsibility for promoting healthy eating for their children [71]. For FBDG to be attainable, it is necessary to consider the cultural context in which they are being developed [102].

Future studies should assess the cultural context in terms of meal dietary patterns, and where and when the foods are consumed [59,102]. Given that children and adolescents spend a considerable part of the day at school [103], this environment plays a key role in the food consumption outside of the home for this age group. Although school feeding programs in developing countries promote access to high quality food in public schools, the same does not necessarily occur in private schools [82,104,105]. Moreover, as discussed by Azevedo et al. [106] and Andrade et al. [82] in Brazil, unhealthy foods and sugary beverages may be sold in commercial spaces inside or around schools, including snack bars, suggesting the need for implementing laws to regulate commerce and food publicity in these locations. It is also important to improve the dietary assessment methods to better capture food consumption information, especially when using FFQ.

## 5. Conclusions

Children and adolescents with high parental education tended to have a healthier diet; higher income was inversely associated with “unhealthy” DP in cross-sectional studies in HHDC. In MHDC, no association between education or income and DPs was found, although greater adherence to “unhealthy” DP was observed in children and adolescents with a higher income/education and with a lower income/education, suggesting the effects of nutritional transition in these countries.

## Figures and Tables

**Table 1:** Association between socioeconomic status and dietary patterns in children and adolescents in cohort studies.

Country	Survey Year/Follow up Time Points	Study Location	HDI	Age Range	Sample Size	DAM/DPM	SES Indicator	DP Identified	Association of SES with DP	Reference
High Human Development Countries										
England	1991–1992/2004–2005 7, 10 and 13 years	Avon	0.87 (2000)	7–13	6202 (7 years) 5949 (10 years) 4986 (13 years)	3-day UFD/RRR	Maternal education	(a) “Energy dense, high fat, low fiber”	(a) Inverse (boys) (a) Data not shown (girls)	[21]
England	1991–1992/2004–2005 7, 10 and 13 years	Avon	0.87 (2000)	7–13	6837 (7 years) 6972 (10 years) 5661 (13 years)	3-day FD and 24-h DR/CA	Maternal education	(a) “Processed” cluster at all 3 time points (*n* = 240) (b) “Processed” cluster at any 2 time points (*n* = 692)	(a) Inverse (b) Inverse	[24]
								(c) “Healthy” cluster at all 3 time points (*n* = 714) (d) “Healthy”” cluster at any 2 time points (*n* = 353)	(c) Positive (d) Positive	
Belgium, Cyprus Estonia GermanyHungary, Italy Spain Sweden	2007–2008/2009–2010 2–9 years 4–11 years	Multi-center	0.87 * (2010)	2–9 to 4–11	9301 4678 B 4623 G	FFQ/CA	Maternal education Paternal education Family income	(a) “Processed” cluster at 2 time points (*n* = 2046) (b) “Sweet” cluster at 2 time points (*n* = 1300) (c) “Healthy” cluster at 2 time points (*n* = 1300) (d) “Processed/sweet” cluster at baseline, “healthy” cluster at follow-up (*n* = 2289) (e) “Healthy” cluster at baseline, “processed/sweet” cluster at follow-up (*n* = 445)	(a) None (maternal education and income) (a) Inverse (paternal education) (b) Inverse (maternal and paternal education and income) (c) Positive (maternal and paternal education, income) (d) None (maternal education and income) (d) positive (paternal education) (e) None (maternal and paternal education) (e) Inverse (income)	[28]
France	2002–2007 2,3 and 5 years	National	0.85 (2000)	2–5	989 527 B 462 G	3-day FFQ/PCA	Maternal education and family income	Multi–time point dietary patterns spanning 2–5 years:		[26]
								(a) “Processed and fast foods”	(a) Inverse (maternal education)	
									(a) None (income)	
								(b) “Guidelines adherence”	(b) Positive (maternal education)	
									(b) None (income)	
France	2002–2007 2,3 and 5 years	National	0.85 (2000)	2–5	974 520 B 454 G	3-day FFQ/PCA	Paternal education	Multi–time point dietary patterns spanning 2–5 years:		[63]
								(a) “Processed and fast foods”	(a) None	
								(b) “Guidelines adherence”	(b) Positive	
Korea	2009–2015/7 and 9 years	Seoul	0.88 (2010)	7–9	279 (7 years) 360 (9 years)	FFQ/PCA	household income	(a) “Healthy intake”	(a) None	[42]
								(b) “Animal food intake”	(b) None	
								(c) “Snack intake”	(c) None	
Medium Human Development Countries										
Brazil	2004–2008 2 and 4 years	Pelotas	0.70 (2010)	24 to 48 months	3790 (24 months) 3714 (48 months)	A list of food items consumed in the 24 h of the last day previous to the interview that the child at as usual/PCA	Maternal education	24 months		[29]
								(a) “Staple”	(a) Inverse	
								(b) “Milks”	(b) None	
								(c) “Snack”	(c) Inverse	
								(d) “Beverage”	(d) None	
								(e) “Meat and vegetables”	(e) Positive	
								48 months		
								(f) “Milks”	(f) Positive	
								(g) “Staple”	(g) Inverse	
								(h) “Beverages”	(h) Positive	
								(i) “Snack”	(i) Inverse	
								(j) “Treats”	(j) Inverse	

B, boys; G, girls; CA, cluster analysis; DAM, dietary assessment method; DPM, dietary pattern method; 24-h DR, 24-h dietary recall; FA, factor analysis; FD, food diary; FFQ, food frequency questionnaires; PCA, principal component analysis; RRR, reduced rank regression; UFD, unweighted food diary. * Average HDI for included countries in the article.

**Table 2:** Association between socioeconomic status and dietary patterns in children and adolescents from High Human Development Countries in cross-sectional studies or longitudinal studies with cross-sectional analysis.

Country	Survey Year	Study Location	HDI	Age Range	Sample Size	DAM/DPM	SES Indicator	DP Identified	Association of SES with DP		Reference
Norway	2007	County	0.94 (2010)	9–10	924 461 B 463 G	FFQ/PCA	Parent’s education	(a) “Snacking” (b) Junk/convenient” (c) Varied Norwegian” (d) “Dieting”	(a) Inverse (b) U shaped (c) None (d) None		[40]
Australia	2007	National	0.93 (2010)	2–8	2287 1166 B 1121 G	2-day, 24-h DR/PCA	Parent’s education	(a) “Healthy, meat and vegetable”	(a) Positive		[43]
								(b) “Combination”	(b) Positive		
Australia	2008	National	0.93 (2010)	12–18	764 397 B 367 G	FFQ/PCA	Family income	(a) “Fruit, salad, cereals, and fish”	(a) None		[23]
								(b) “High fat and sugar”	(b) None		
								(c) “Vegetables”	(c) None		
Australia	2003–2004	Perth	0.90 (2000)	14	1613 826 B 787 G	FFQ/FA	Maternal education and family income	(a) “Healthy”	(a) Positive (maternal education)		[27]
									(a) None (income)		
								(b) “Western”	(b) None (maternal education)		
									(b) Inverse (income)		
Scotland	2006	National	0.91 (2010)	5–17	1233	FFQ/PCA	Main food provider education and family income	5–11-year-old			[44]
									Boys	Girls	
					5–11 years old (381 B, 340 G) 12–17 years old (250 B, 262 G)			(a) “Fruit and vegetables”	(a) None (education)	(a) Positive (education, income)	
									(a) Positive (income)		
								(b) “Snacks”	(b) Inverse (education, income)	(b) None (education)	
										(b) Inverse (income)	
								(c) “Fish and sauce”	(c) None (education, income)	(c) NA	
								(d) “Puddings”	(d) NA	(d) Positive (education, income)	
					12–17 years old (250 B, 262 G)			12–17 years old			
									Boys	Girls	
								(e) “Vegetables”	(e) positive (education, income)	(e) Positive (education, income)	
								(f) “Fruits”	(f) NA	(f) None (education, income)	
								(g) “Puddings”	(g) None (education)	(g) Inverse (education, income)	
									(g) Inverse (income)		
								(h) “Starchy food and drinks”	(h) None (education, income)	(h) NA	
Spain	2007–2008	Balearic Islands	0.87	12–17	1231 574 B 657 G	FFQ and 24-h DR/PCA	Parent’s education	(a) “Western” (b) “Mediterranean” (c) “Dairy products” (d) “Fast food and sweets”	(a) None (b) None (c) Positive (d) Inverse		[45]
Spain	1998–2000	National	0.83 (2000)	2–24	3534 1629 B 1905 G	FFQ and 24-h DR/PCA	Maternal or parental education	(a) “Snacky”	(a) Positive		[64]
								(b) “Healthy”	(b) Positive		
Canada	2004	National	0.87 (2000)	2–18	10,038 5119 B 4919 G	1-day, 24-h DR/CA	Household education and family income	2–5 years old			[53]
									Boys	Girls	
								(a) “Fruit drink” (*n* = 315)	None (education and income)		
								(b) “Fruit juice” (*n* = 320)			
								(c) “Milk” (*n* = 422)			
								(d) “High fat milk” (*n* = 268)			
								(e) “Moderate” (*n* = 825)			
								6–11 years old			
									Boys	Girls	
								(f) “Soft drink” (*n* = 412)	High fat milk “was more frequent in lower income None (education)	None (education, income)	
								(g) “Fruit drink” (*n* = 601)			
								(h) “Fruit juice” (*n* = 398)			
								(i) “Milk” (*n* = 670)			
								(j) “High fat milk” (*n* = 283)			
								(l) “Moderate” (*n* = 1249)			
								12–18 years old			
									Boys	Girls	
								(m) “Soft drink” (*n* = 648)	None (education, income)	None (education, income)	
								(n) “Fruit drink” (*n* = 701)			
								(o) “Milk” (*n* = 783)			
								(p) “Moderate” (*n* = 2143)			
England	1991–1992/1998–1999	Avon	0.87 (2000)	7	6056 3131 B 2925 G	FFQ/CA	Maternal education	(a) “Processed” (*n* = 4177)	(a) Inverse		[20]
								(b) “Plant based” (*n* = 2065)	(b) Positive		
								(c) “Traditional British” (*n* = 2037)	(c) None		
England	1991–1992/2004–2006	Avon	0.87 (2000)	13	3951 1916 B 2035 G	FFQ/PCA	Maternal education	(a) “Traditional/health-conscious”	(a) Positive		[46]
								(b) “Processed”	(b) Inverse		
								(c) “Snacks/sugared drinks”	(c) Inverse		
								(d) “Vegetarian”	(d) Positive		
England	1998–1999	Avon	0.87 (2000)	4 and 7	4 years old (6592) 3411 B 3171 G	FFQ/PCA	Maternal education	4 years old			[65]
								(a) “Junk”	(a) Inverse		
								(b) “Health conscious”	(b) Positive		
								(c) “Traditional”	(c) None		
					7 years old (6215) 3196 B 3019 G			7 years old			
								(a) “Junk”	(a) Inverse		
								(b) “Health conscious”	(b) Positive		
								(c) “Traditional”	(c) None		
Greece	2007–2011	Creete	0.87 (2000)	4	683	FFQ/PCA	Parent’s education	(a) “Mediterranean (b) “Snacky” (c) “Western””	(a) None (b) Inverse (c) None		[66]
New Zealand	1995–1997 2002–2004	Auckland	0.87 (2000)	3.5–7	550 (3.5 years); 591 (7 years)	FFQ/PCA	Maternal education	(a) “Junk”	(a) None		[67]
								(b) “Traditional”	(b) None		
								(c) “Healthy”	(c) None		
Finland	2003–2005	Oulu and Tampere	0.86 (2000)	3 and 6	3 years old (708)	3-day FD/CA	Maternal education	3 years old			[68]
								(a) “Fast food, sweet” (*n* = 387)	(a) Inverse		
					6 years old (841)			6 years old			
								(a) “Fast food, sweet” (*n* = 198)	(a) None		
Portugal	2009–2010	Porto	0.82 (2010)	4	3422 1749 B 1673 G	FFQ/LCA	Maternal education Family income	(a) Energy-dense foods dietary pattern (*n* = 1400) (b) Snacking (*n* = 484) (c) Healthier (*n* = 1538)	(a) Inverse (education) (a) None (income) (b) Inverse (education) (b) None (income) (c) NA		[69]
Portugal	2006–2007	Porto	0.82 (2010)	5–10	1976 985 B 991 G	FFQ/PCA	Maternal education	(a) “Vegetables, pulses, fruit, olive oil”	(a) Positive		[49]
								(b) “Fish, meat, processed meats, eggs, and starchy foods”	(b) Positive		
								(c) “Vegetable soup, olive oil, butter, starchy foods, and bread”	(c) Positive		
								(d) “Fast-food, SSB, and pastry”	(d) Inverse		
European cities	2006–2007	Athens, Dortmund, Ghent, Lille, Rome, Stockholm, Vienna, and Zaragoza	Mean 0.81 (2010)	12.5–17.5	2213 1021 B 1192 G	24-h DR HELENA-Dietary Assessment Tool (DIAT)/PCA	Parent’s education	Boys (a) “Western” (b) Traditional European (c) Breakfast Girls (d) “Western” (e) Traditional European (f) Breakfast (g) Monotonous	Boys (a) Inverse (maternal educational) (a) None (paternal education) (b) Positive (parent’s education) (c) Positive (parent’s education) Girls (d) Inverse (parent’s education) (e) Positive (parent’s education) (f) None (parent’s education) (g) None (parent’s education)		[47]
Australia	2011–2013	Adelaide	0.94 (2010)	9–11	508 236 B 272 G	FFQ/PCA	Household income and parent’s education.	(a) Unhealthy (b) Healthy	(a) Inverse (income and parent’s education) (b) None (income and parent’s education)		[70]
Canada	2011–2013	Ottawa	0.89 (2010)	9–11	551 230 B 321 G	FFQ/PCA	Household income and parent’s education.	(a) Unhealthy (b) Healthy	(a) Inverse (income and parent’s education) (b) Positive (income) (b) None (parent’s education)		[70]
Finland	2011–2013	Helsinki, Espoo and Vantaa)	0.87 (2010)	9–11	495 235 B 260 G	FFQ/PCA	Household income and parent’s education.	(a) Unhealthy (b) Healthy	(a) Inverse (income and parent’s education) (b) None (income and parent’s education)		[70]
USA	2011–2013	Baton Rouge	0.90 (2010)	9–11	588 254 B 334 G	FFQ/PCA	Household income and parent’s education.	(a) Unhealthy (b) Healthy	(a) Inverse (income and parent’s education) (b) None (income and parent’s education)		[70]
Portugal	2011–2013	Porto	0.82 (2010)	9–11	667 294 B 373 G	FFQ/PCA	Household income and parent’s education.	(a) Unhealthy (b) Healthy	(a) Inverse (income and parent’s education) (b) Positive (income) (b) None (parent’s education)		[70]
United Kington	2011–2013	Bath and North East Somerset)	0.85 (2010)	9–11	465 208 B 257 G	FFQ/PCA	Household income and parent’s education.	(a) Unhealthy (b) Healthy	(a) Inverse (parent’s education) (a) None (income) (b) Positive (parent’s education) (b) None (income)		[70]
Poland	2010–2011	Central and north-eastern Poland	0.80 (2010)	13–18	1176 551 B 625 G	FFQ/CA	Parent’s education	(a) Low-Fiber” DP (*n* = 446) (b) “Average-Fiber” DP (*n* = 286) (c) “High-Fiber” DP (*n* = 444)	(a, b) NA (c) Positive (parent’s education)		[48]

B, boys; G, girls; CA, cluster analysis; DAM, dietary assessment method; DPM, dietary pattern method; 24-h DR, 24-h dietary recall; FA, factor analysis; FD, food diary; FFQ, food frequency questionnaires; NA, not applicable; PCA, principal component analysis; LCA, latent class analysis.

**Table 3:** Association between socioeconomic status and dietary patterns in children and adolescents from Medium Human Development Countries and Low Human Development Countries in cross-sectional studies or longitudinal studies with cross-sectional analysis.

Country	Survey Year	Study Location	HDI	Age Range	Sample Size	DAM/DPM	SES Indicator	DP Identified	Association of SES with DP	Ref.
Medium Human Development Countries										
Portugal	2003–2004	Porto	0.78 (2000)	13	1489 687 B 802 G	FFQ/CA	Parent’s education	(a) “Healthier” (*n* = 239) (b) “Dairy products” (*n* = 442) (c) “Fast food and sweets” (*n* = 212)	(a) Positive (b) Positive (c) Inverse	[38]
Malaysia	2014	District Selangor	0.78 (2014)	13–17	2480 882 B 1366 G	FFQ/PCA	Parent’s education and family income	(a) “Fruit and vegetable” (b) “Sugar and fat” (c) “Meat and Chicken”	(a) Positive (education) (a) None (income) (b) Positive (education) (b) None (income) (c) Positive (education, income)	[32]
Malaysia	-	Kelantan	0.78 (2014)	12–19	454 204 B 250 G	FFQ/PCA	Parent’s education and family income	(a) “Western-based” (b) “Health-based” (c) “Local-based”	Malay adolescents (a) Inverse (income) (a) None (maternal and paternal education) (b) None ((maternal and paternal education and income) (c) None ((maternal and paternal education and income) **Chinese adolescents** (a) None ((maternal and paternal education and income) (b) Positive (maternal education) (b) None (paternal education and income) (c) None (maternal and paternal education and income)	[41]
England	1991–1992 1994–1995	Avon	0.77 (1990)	3	7814 4019 B 3795 G	FFQ/PCA	Maternal education	(a) “Junk”	(a) Inverse	[22]
								(b) “Healthy”	(b) Positive	
								(c) “Traditional”	(c) Positive	
								(d) “Snacks”	(d) Positive	
England	1993–1994	Avon	0.77 (1990)	2	9599 4963 B 4636 G	FFQ/PCA	Maternal education	(a) “Family foods” (b) “Sweet and easy” (c) “Healthy conscious”	(a) Positive (b) Inverse (c) Positive	[25]
Brazil	2008	Salvador	0.70 (2010)	7–14	1136 577 B 559 G	FFQ/PCA	Maternal education and family income	(a) “Obesogenic” (b) “Traditional”	(a) Positive (education, income) (b) None (education, income)	[31]
Brazil	2009–2010	Diamantina	0.70 (2010)	5	232 -	FFQ/PCA	Maternal education and per capita income	(a) “Mixed diet” (b) “Snack” (c) “Unhealthy”	(a) None (education) (a) Positive (income) (b) Positive (education) (b) Inverse (income) (c) None (education) (c) Positive (income)	[30]
Brazil	2011	Montes Claros	0.70 (2010)	11–17	535 171 B 364 G	FFQ/PCA	Parent’s education and family income	(a) “Junk food” (b) “Healthy” (c) “Traditional”	(a) None (education) (a) Positive (income) (b) None (education, income) (c) None (education, income)	[33]
Brazil	2012–2013	Viçosa	0.70 (2010)	8–9	328 135 B 193 G	3-day FD PCA	Maternal education	(a) “Sweetened drinks and snacks” (b) “Egg-dairy”	(a) Positive (b) Positive	[52]
Brazil	2008–2009	National	0.70	12.5–17.5	3194 1635 B 1559 G	2-day FD/PCA	Maternal education and family income	Boys (a) “Traditional Brazilian” (b) “Western” (c) “Snacks” (d) “Healthy” Girls (e) “Western” (f) “Breakfast” (g) “Sweets and Fried Foods” (h) “Traditional Brazilian”	Boys (a) Positive (maternal education and income) (b) Positive (maternal education and income) (c) None (income) (c) None (maternal education) (d) None (maternal education and income) Girls (e) Positive (income and maternal education) (f) None (income and maternal education) (g) None (income and maternal education) (h) Inverse (income) h) None (maternal education)	[47]
Brazil	2014	Campinas	0.70 (2010)	2–9	929	FFQ/FA	Maternal education and family income	(a) “Traditional” (b) “Ultraprocessed”	NA (a) Inverse maternal education (b)None (family income)	[71]
India	1997–1998 2006–2007	Mysore	0.52 (2010)	9.5	538 254 B 284 G	FFQ PCA	Parent’s education	(a) “Snack and fruit”	(a) None	[50]
								(b) “Lacto-vegetarian”	(b) None	
Lebanon	2011–2012	National	0.77 (2014)	2–5	525 281 B 244 G	24-h DR/FA	Maternal education	(a) Fast food and Sweets (b) Traditional Lebanese	(a) Inverse (maternal education) (b) Positive (maternal education)	[72]
China	2009	Beijing and four provincial capital cities including Haerbin, Jinan, Shanghai, and Guangzhou	0.66 (2010)	6–13	5267 2643 B 2624 G	24-h DR/FA and CA	Parent’s education and monthly household income	(a) “Healthy” (*n* = 3679) (b) “Transitive diet” (high positive loadings on organ meat, pork, seafood, processed meat, edible fungi and algae and light vegetables) (*n* = 1395) (c) “Western” (m = 193)	High Healthy DP was more frequent in lower parent’s education and High “transitive diet” and “western” DP was more frequent in higher parent’s education. High transitive diet was more frequent in higher income	[51]
China	2010	Taiwan	0.66 (2010)	5	18046 9463 B 8583 G	FFQ/PCA	Parent’s education and family monthly income	(a) Unhealthy non-core food (b) “Health-conscious food”	(a) Inverse (parent’s education and income) (b) Positive (parent’s education and income)	[60]
Brazil	2011–2013	São Paulo	0.70 (2010)	9–11	501 245 B 256 G	FFQ/PCA	Household income and parent’s education	(a) Unhealthy (b) Healthy	(a) Inverse (parent’s education) (a) None (income) (b) None (income and parent’s education)	[70]
Colombia	2011–2013	Bogota	0.69 (2010)	9–11	914 454 B 460 G	FFQ/PCA	Household income and parent’s education	(a) Unhealthy (b) Healthy	(a) None (income and parent’s education) (b) None (parent’s education) (b) Positive (income)	[70]
China	2011–2013	Tianjin	0.66 (2010)	9–11	542 288 B 254 G	FFQ/PCA	Household income and parent’s education	(a) Unhealthy (b) Healthy	(a) None (income and parent’s education) (b) None (parent’s education and income)	[70]
South Africa	2011–2013	Cape Town	0.60 (2010)	9–11	423 167 B 256 G	FFQ/PCA	Household income and parent’s education	(a) Unhealthy (b) Healthy	(a) Inverse (income and parent’s education) (b) None (parent’s education and income)	[70]
India	2011–2013	Bangalore	0.52 (2010)	9–11	602 282 B 320 G	FFQ/PCA	Household income and parent’s education	(a) Unhealthy (b) Healthy	(a) Inverse (income) (a) None (parent’s education) (b) Positive (parent’s education and income)	[70]
Low Human Development Country												
Kenya	2011–2013	Nairobi	0.47 (2010)	9–11	552 257 B 295 G	FFQ/PCA	Household income and parent’s education	(a) Unhealthy (b) Healthy	(a) Inverse (income and parent’s education) (b) None (parent’s education and income)	[70]

B, boys; G, girls; DAM, dietary assessment method; DPM, dietary pattern method; 24-h DR, 24-h dietary recall; FA, factor analysis; FD, food diary; FFQ, food frequency questionnaires; PCA, principal component analysis.

**Table 4:** Direction of association between socioeconomic status and unhealthy, healthy and traditional DPs in children and adolescents by the study design and the level of human development.

Study Design/Level of Human Development	SES Indicator x Dietary Patterns	Total (Number of Times the Association Was Tested)	Direction of Association *n* (%)		
			Positive	Inverse	No Association
Cohort/HHDC	Education x Unhealthy	11	0	7 (63.6)	4 (36.4)
	Education x Healthy	8	7 (87.5)	0	1 (12.5)
	Education x Traditional	0	0	0	0
	Income x Unhealthy	4	0	2 (50.0)	2 (50.0)
	Income x Healthy	4	1 (25.0)	0	3 (75.0)
	Income x Traditional	0	0	0	0
Cohort/MHDC	Education x Unhealthy	3	0	3 (100.0)	0
	Education x Healthy	1	1 (100.0)	0	0
	Education x Traditional	2	0	2 (100.0)	0
	Income x Unhealthy	0	0	0	0
	Income x Healthy	0	0	0	0
	Income x Traditional	0	0	0	0
Cross-sectional/HHDC	Education x Unhealthy	32	2 (9.3)	22 (68.8)	7 (21.9)
	Education x Healthy	22	12 (54.5)	0	10 (45.5)
	Education x Traditional	8	3 (37.5)	0	5 (62.5)
	Income x Unhealthy	15	1(6.6)	10 (66.7)	4 (26.7)
	Income x Healthy	14	6 (42.9)	0	8 (57.1)
	Income x Traditional	0	0	0	0
Cross-sectional/MHDC	Education x Unhealthy	27	8 (29.6)	8 (29.6)	11 (40.8)
	Education x Healthy	17	7 (41.2)	1 (5.9)	9 (52.9)
	Education x Traditional	12	4 (33.3)	0	8 (66.7)
	Income x Unhealthy	19	5 (26.3)	5 (26.3)	9 (47.4)
	Income x Healthy	12	3 (25.0)	0	9 (75.0)
	Income x Traditional	7	2 (28.6)	1 (14.3)	4 (57.1)
Cross-sectional/LHDC	Education x Unhealthy	1	0	1 (100.0)	0
	Education x Healthy	1	0	0	1 (100.0)
	Education x Traditional	0	0	0	0
	Income x Unhealthy	1	0	1 (100.0)	0
	Income x Healthy	1	0	0	1 (100.0)
	Income x Traditional	0	0	0	0

## Supplementary Materials

The following are available online at http://www.mdpi.com/2072-6643/10/4/436/s1. Table S1: PRISMA 2009 Checklist; Table S2: Database search strategy; Table S3: Summary of characteristics of the dietary assessment methods of the studies included in the systematic review; Table S4: Risk of bias assessed by Meta Analysis of Statistics Assessment and Review Instrument (MAStARI) critical appraisal tools. Risk of bias was categorized as High when the study reaches up to 49% score “yes”, Moderate when the study reached 50% to 69% score “yes”, and Low when the study reached more than 70% score “yes”.
