# Supplementary material for: Dietary Patterns of Children and Adolescents from High, Medium and Low Human Development Countries and Associated Socioeconomic Factors: A Systematic Review
Source: Nutrients. 2018 Mar 30;10(4):436. doi: 10.3390/nu10040436 (PMC5946221; doi:10.3390/nu10040436)
Supplement: Supplementary file 1 [file nutrients-10-00436-s001.pdf]

Table S1. PRISMA 2009 Checklist.

Section/topic	#	Checklist item	Reported on page #
**TITLE**			
Title	1	Identify the report as a systematic review, meta-analysis, or both.	1
**ABSTRACT**			
Structured summary	2	Provide a structured summary including, as applicable: background; objectives; data sources; study eligibility criteria, participants, and interventions; study appraisal and synthesis methods; results; limitations; conclusions and implications of key findings; systematic review registration number.	1
**INTRODUCTION**			
Rationale	3	Describe the rationale for the review in the context of what is already known.	2
Objectives	4	Provide an explicit statement of questions being addressed with reference to participants, interventions, comparisons, outcomes, and study design (PICOS).	2
**METHODS**			
Protocol and registration	5	Indicate if a review protocol exists, if and where it can be accessed (e.g., Web address), and, if available, provide registration information including registration number.	2
Eligibility criteria	6	Specify study characteristics (e.g., PICOS, length of follow-up) and report characteristics (e.g., years considered, language, publication status) used as criteria for eligibility, giving rationale.	Implied in "Inclusion Criteria" (page 3)
Information sources	7	Describe all information sources (e.g., databases with dates of coverage, contact with study authors to identify additional studies) in the search and date last searched.	4 and Figure 1
Search	8	Present full electronic search strategy for at least one database, including any limits used, such that it could be repeated.	Online Supplemental

			Material 1
Study selection	9	State the process for selecting studies (i.e., screening, eligibility, included in systematic review, and, if applicable, included in the meta-analysis).	Implied in “Study selection” (page 3)
Data collection process	10	Describe method of data extraction from reports (e.g., piloted forms, independently, in duplicate) and any processes for obtaining and confirming data from investigators.	3,4
Data items	11	List and define all variables for which data were sought (e.g., PICOS, funding sources) and any assumptions and simplifications made.	3,4
Risk of bias in individual studies	12	Describe methods used for assessing risk of bias of individual studies (including specification of whether this was done at the study or outcome level), and how this information is to be used in any data synthesis.	Implied in “Risk of bias within individual studies” (page 5)
Summary measures	13	State the principal summary measures (e.g., risk ratio, difference in means).	Implied in “Data items” (page 4)
Synthesis of results	14	Describe the methods of handling data and combining results of studies, if done, including measures of consistency (e.g., I^2) for each meta-analysis.	Implied in “Data items” (page 4)

Table Table S1. *Cont.*

**Section/topic**	**#**	**Checklist item**	**Reported on page #**
Risk of bias across studies	15	Specify any assessment of risk of bias that may affect the cumulative evidence (e.g., publication bias, selective reporting within studies).	Implied in "Risk of bias within individual studies" (page 5)
Additional analyses	16	Describe methods of additional analyses (e.g., sensitivity or subgroup analyses, meta-regression), if done, indicating which were pre-specified.	Subgroup: HHDC, MHDC and LHDC (tables 1, 2, 3 and 4, pages 8-21 and in "Synthesis of results) – page 22-24
**RESULTS**			
Study selection	17	Give numbers of studies screened, assessed for eligibility, and included in the review, with reasons for exclusions at each stage, ideally with a flow diagram.	Page 5 and Figure 1
Study characteristics	18	For each study, present characteristics for which data were extracted (e.g., study size, PICOS, follow-up period) and provide the citations.	Pages 5, 22 and tables 1, 2,3
Risk of bias within studies	19	Present data on risk of bias of each study and, if available, any outcome level assessment (see item 12).	Page 22 and Online Supplemental Material 2 and 3
Results of individual studies	20	For all outcomes considered (benefits or harms), present, for each study: (a) simple summary data for each intervention group (b) effect estimates and confidence intervals, ideally with a forest plot.	Tables 1, 2 and 3
Synthesis of results	21	Present results of each meta-analysis done, including confidence intervals and measures of	not applicable

		consistency.	
Risk of bias across studies	22	Present results of any assessment of risk of bias across studies (see Item 15).	Page 22 and online Supplemental Material 2 and 3
Additional analysis	23	Give results of additional analyses, if done (e.g., sensitivity or subgroup analyses, meta-regression [see Item 16]).	Table 4
**DISCUSSION**			
Summary of evidence	24	Summarize the main findings including the strength of evidence for each main outcome; consider their relevance to key groups (e.g., healthcare providers, users, and policy makers).	25
Limitations	25	Discuss limitations at study and outcome level (e.g., risk of bias), and at review-level (e.g., incomplete retrieval of identified research, reporting bias).	26 and 27
Conclusions	26	Provide a general interpretation of the results in the context of other evidence, and implications for future research.	25, 26 and 27
**FUNDING**			
Funding	27	Describe sources of funding for the systematic review and other support (e.g., supply of data); role of funders for the systematic review.	27

From: Moher D, Liberati A, Tetzlaff J, Altman DG, The PRISMA Group (2009). Preferred Reporting Items for Systematic Reviews and Meta-Analyses: The PRISMA Statement. PLoS Med 6(7): e1000097. doi:10.1371/journal.pmed1000097

Table S2. Database search strategy.

**Database**	**Search** (Jan 22th, 2018)
**EMBASE**	(child OR children OR schoolchildren OR preschool OR preschoolers OR pediatrics OR pediatric OR paediatric OR adolescent OR adolescents OR adolescence OR childhood OR teen OR teens OR teenager OR teenagers OR youth OR youths)<br>**AND**<br>("socioeconomic factors" OR "socioeconomic factor" OR "socio-economic factors" OR "socio-economic factor" OR "socioeconomic status" OR education OR "educational status" OR "parent education" OR "parents education" OR income OR "maternal schooling")<br>**AND**<br>(diet OR diets OR "food consumption" OR "food habit" OR "food habits" OR "feeding behaviour" OR "feeding behavior" OR "feeding behaviors" OR "feeding behaviours" OR "dietary pattern" OR "dietary patterns" OR "diet pattern" OR "diet patterns" OR "eating pattern" OR "eating patterns" OR "dietary behavior" OR "dietary behaviors" OR "dietary behaviour" OR "dietary behaviours" OR "feeding pattern" OR "feeding patterns" OR "eating behavior" OR "eating behaviors"OR "eating behaviour" OR "eating behaviours")<br>**AND**<br>("principal component analysis" OR "cluster analysis" OR "cluster analyses" OR "reduced rank regression" OR "factor analysis" OR "factor analyses" OR "treelet transform" OR "latent class analysis")

**LILACS**	“crianças” OR "crianca" OR "nino" OR "ninos" OR “pré-escolar” OR “pré-escolares” OR “preescolar” OR "adolescente" OR "adolescentes" OR "adolescencia" OR "infancia" OR "escolar" OR "escolares" OR "estudiante" OR "estudiantes") [palavras]<br>**AND**<br>"fatores socioeconomicos" OR "factores socioeconomicos" OR "condicoes socioeconomicas" OR "condiciones socioeconomicas" OR "educacao" OR "educacion" OR "escolaridade" OR "escolaridad" OR "escolaridade materna" OR "renda" OR "renda familiar" OR "renta" OR "renta familiar") [palavras]<br>**AND**<br>“dieta” OR “dietas” OR “consumo de alimentos” OR “consumo alimentar” OR "padroes alimentares" OR "comportamento alimentar" OR "conducta alimentaria" OR "patrones alimentarios" [palavras]
**PubMed**	("child"[MeSH Terms] OR "child"[All Fields] OR "children"[All Fields] OR schoolchildren[All Fields] OR preschool[All Fields] OR "child, preschool"[MeSH Terms] OR "preschool child"[All Fields] OR "preschoolers"[All Fields] OR "pediatrics"[MeSH Terms] OR "pediatrics"[All Fields] OR "pediatric"[All Fields] OR "paediatric"[All Fields] OR "adolescent"[MeSH Terms] OR "adolescent"[All Fields] OR "adolescents"[All Fields] OR "adolescence"[All Fields] OR "childhood"[All Fields] OR "teen"[All Fields] OR "teens"[All Fields] OR "teenager"[All Fields] OR "teenagers"[All Fields] OR "youth"[All Fields] OR "youths"[All Fields])<br>**AND**<br>("socioeconomic factors"[MeSH Terms] OR "socioeconomic factors"[All Fields] OR "socioeconomic factor"[All Fields] OR "socio-economic factors"[All Fields] OR "socio-economic factor"[All Fields] OR "socioeconomic status"[All Fields] OR "education"[All Fields] OR "educational status"[MeSH Terms] OR "educational status"[All Fields] OR "education"[MeSH Terms] OR "parent education"[All

	Fields] OR "parents education"[All Fields] OR "income"[MeSH Terms] OR "income"[All Fields] OR "maternal schooling"[All Fields])<br>**AND**<br>("diet"[MeSH Terms] OR "diet"[All Fields] OR "diets"[All Fields] OR "food consumption"[All Fields] OR "food habit"[All Fields] OR "food habits"[MeSH Terms] OR "food habits"[All Fields] OR "feeding behaviour"[All Fields] OR "feeding behavior"[MeSH Terms] OR "feeding behavior"[All Fields] OR "feeding behaviors"[All Fields] OR "feeding behaviour"[All Fields] OR "feeding behaviours"[All Fields] OR "dietary pattern"[All Fields] OR "dietary patterns"[All Fields] OR "diet pattern"[All Fields] OR "diet patterns"[All Fields] OR "eating pattern"[All Fields] OR "eating patterns"[All Fields] OR "dietary behavior"[All Fields] OR "dietary behaviors"[All Fields] OR "dietary behaviour"[All Fields] OR "dietary behaviours"[All Fields] OR "feeding pattern"[All Fields] OR "feeding patterns"[All Fields] OR "eating behavior"[All Fields] OR "eating behaviors" [All Fields] OR "eating behaviour" [All Fields] OR "eating behaviours" [All Fields])<br>**AND**<br>("principal component analysis"[MeSH Terms] OR "principal component analysis"[All Fields] OR "cluster analysis"[MeSH Terms] OR "cluster analysis"[All Fields] OR "cluster analyses"[All Fields] OR "reduced rank regression"[All Fields] OR "factor analysis, statistical"[MeSH Terms] OR "statistical factor analysis"[All Fields] OR "factor analysis"[All Fields] OR "factor analyses"[All Fields] OR "treelet transform"[All Fields] OR "latent class analysis"[All Fields])
**Science Direct**	(child* OR schoolchildren OR preschool* OR adolescen* OR teen* OR youth*)<br>**AND**

	("socioeconomic factor*" OR “socio-economic factor*” OR “socioeconomic status” OR education* OR “educational status” OR “parent* education*” OR income OR "maternal schooling")<br>**AND**<br>(diet* OR “food consumption” OR “food habit*” OR "feeding behavio*" OR "dietary pattern*" OR “diet pattern*” OR "eating pattern*" OR "dietary behavio*" OR "feeding pattern*" OR "eating behavio*")
**Scopus**	(child OR schoolchildren OR childhood OR preschool OR adolescent OR adolescence OR teen OR teenager OR youth)<br>**AND**<br>("socioeconomic factor" OR “socio-economic factor” OR “socioeconomic status” OR education OR "educational status" OR “parent education” OR income OR "maternal schooling")<br>**AND**<br>(diet OR “food consumption” OR “food habit” OR "feeding behavior" OR "feeding behaviour" OR "dietary pattern" OR “diet pattern” OR “eating pattern" OR "dietary behavior" OR "dietary behaviour" OR "feeding pattern" OR "eating behavior")<br>**AND**<br>("Principal Component Analysis" OR "Cluster Analysis" OR "cluster analyses" OR "reduced rank regression" OR "factor analysis" OR "factor analyses" OR "treelet transform" OR “latent class analysis”)
**Web of Science**	(child* OR schoolchildren OR preschool* OR adolescen* OR teen* OR youth*)<br>**AND**

	("socioeconomic factor*" OR “socio-economic factor*” OR “socioeconomic status” OR education* OR “educational status” OR “parent* education*” OR income OR "maternal schooling")<br>**AND**<br>(diet* OR “food consumption” OR “food habit*” OR "feeding behavio*" OR "dietary pattern*" OR “diet pattern*” OR "eating pattern*" OR "dietary behavio*" OR "feeding pattern*" OR "eating behavio*")<br>**AND**<br>("Principal Component Analys*" OR "Cluster Analys*" OR "reduced rank regression" OR "factor analys*" OR "treelet transform" OR “latent class analys*”)
	**Grey literature**
**Google Scholar**	(children OR preschool OR adolescent) AND ("socioeconomic factors" OR education OR income) AND (diet OR diets OR food OR feeding OR dietary OR eating) AND ("Principal Component" OR Cluster OR regression OR factor OR "treelet transform" OR "latent class")
**ProQuest**	(diet OR diets OR “food consumption” OR “food habit” OR “food habits” OR "feeding behavior" OR "feeding behaviors" OR "feeding behaviour" OR "feeding behaviours" OR "dietary pattern" OR "dietary patterns" OR “diet pattern” OR “diet patterns” OR "eating pattern" OR "eating patterns" OR "dietary behavior" OR "dietary behaviors" OR "dietary behaviour" OR "dietary behaviours" OR "feeding pattern" OR "feeding patterns" OR "eating behavior" OR "eating behaviors" OR "eating behaviour" OR "eating behaviours")<br>**AND**

	(child OR children OR schoolchildren OR preschool OR preschoolers OR pediatric OR paediatric OR adolescent OR adolescents OR adolescence OR childhood OR teen OR teens OR teenager OR teenagers OR youth OR youths)<br>**AND**<br>("socioeconomic factors" OR "socioeconomic factor" OR "socio-economic factors" OR "socio-economic factor" OR "socioeconomic status" OR "education" OR "educational status" OR "parent education" OR "parents education" OR OR income OR "maternal schooling")<br>**AND**<br>("Principal Component Analysis" OR "Cluster Analysis" OR "cluster analyses" OR "reduced rank regression" OR "factor analysis" OR "factor analyses" OR "treelet transform" OR "latent class analysis")

Table S3. Summary of characteristics of the dietary assessment methods of the studies included in the systematic review.

Author(s) and country	Age, year or month, range (n participants)	Dietary assessment method			Validation study			Total score/ Risk of bias based on the quality of the dietary methodology
		Type (Recall/ report period)	Structure	Reporter	In the population (children or adolescents) living in the same country of the study?	Reference method	Results	
**Cohort studies from High and Medium Human Development Countries**								
Ambrosini et al. [21] England	7y (6,202), 10y (5,949), and 13y (4,986)	UFD (3 non-consecutive days)	NA	7 y (parents) 10 and 13 y (children completed the diary with input from an adult as required)	NA	NA	NA	30 Low risk of bias
Northstone et al.[24] England	7y (6,837), 10y (6,972) and 13y (5,661)	FD	NA	7 y (caregiver) 10 and 13 y (children completed the	NA	NA	NA	30 Low risk of bias

		(3 non-consecutive days)		diary with input from an adult as required)				
Fernández-Alvira et al. [28]<br><br>Belgium, Cyprus, Estonia, Germany, Hungary, Italy, Spain, Sweden	2-9 and 4-11y (9,301)	FFQ<br><br>(Last month)	43 food items. CFC: 8 responses ranging from "Never/less than once per week" to "Four or more times per day", and "I have no idea"<br><br>The FFQ referred to meals outside the school canteen or child care meal provision settings only.	Parents	**Reprod.**<br>Yes, except for Germany and Spain<br><br>**Validity**<br>Yes (validation for the milk consumption frequencies)	Calcium and potassium urinary Concentration	**Reproducibility** (Lanfer et al.,2011)<br><br>Weighted kappa coefficients: 0.23 to 0.68; Spearman's correlation coefficients: 0.32 to 0.76;<br><br>**Validity** (Huybrechts et al., 2011)<br><br>Significant positive correlation between	20 Moderate risk of bias

							milk consumption frequencies and the ratios of uninary calcium (Uca)/urinary creatinine (Uc) (0.16); Weaker but significant positive correlation with the ratios of UCa/Cr (0.07)	
Lioret et al.[26] France	2 and 5y (989)	FFQ (ND)	26 food groups. CFC: 7 responses ranging from ''Never'' to ''Several times per day''	Parents	No	four 24-h DR	**> 10 years** **Reproducibility** ICCs for nutrients: 0.39 for total protein to 0.83 for alcohol. **Validity** De-attenuated Pearson's	20 Moderate risk of bias

							correlation coefficient: 0.25 (dietary fiber) to 0.90 (alcohol).<br><br>Agreement rates (same or adjacent quintile) between 55% (for PUFA) and 95% (for alcohol) Misclassification to an extreme quintile was rare (<5%). (Deschamps et al.,2009)	
Camara et al.[63]<br><br>France	2 and 5y (9,740)	FFQ (ND)	Described in Lioret et al.[26]	Parents	No	four 24-h DR	> 10 years<br>As described by Lioret et al., 2015	20 Moderate risk of bias
Lee et al.[42]<br><br>Korea	279 (7y)<br>360 (9y)	FFQ<br><br>(Past year)	90 food items. CFC: 7 responses ranging from	Parents or guardians	ND	ND	**Reproducibility**<br>(Chung et al., 2015)	

			"rarely eaten" to "more than three times per day". Portion sizes: small, average, or large				Correlation coefficients: 0.5 to 0.8<br><br>**Validity** (Chung et al., 2015) Correlation coefficients: 0.3 to 0.6	15 High risk of bias
Gatica et al. [29]<br><br>Brazil	24 mo (3,790) 48 mo (3,714)	A list of food items or food groups that the child ate as usual<br><br>(Previous day)	The number of times/day each food item was consumed in seven meals or periods of the day: wake-up time, morning, lunch, afternoon, dinner, evening, night but not the amount consumed.	Mother	No	NA	NA	15 High risk of bias
**Cross-sectional studies from High Human Development Countries (HHDC)**								
Oellingrath et al. [40]	9-10y<br><br>(924)	FFQ	39 food items, 11 types of drinks, 13	Parents	No	Not validated	NA	10

Norway		(Last 6 mo)	snack items and 5 main meals. CFC: 7 responses ranging from: 1–3 times a month' to 3 or more times per day'; and 'rarely/never'					High risk of bias
Grieger et al.[43] Australia	2-8y (2,287)	24h DR (2 non-consecutive days)	NA	Child and parents	ND	ND	ND	30 Low risk of bias
McNaughton et al. [23] Australia	12-18y (764)	FFQ (Past year) and 24h DR (One day)	108 foods and beverages items. CFC: 9 responses ranging from: ''never or less than once a month'' to ''6 or more times per day''. Information on	Adolescents	No	WFD	**Adults** (Ireland et al., 1994) The authors described that the FFQ appeared to overestimate the consumption of	25 Moderate risk of bias

			portion sizes was not included.				fruit and vegetable	
Ambrosini et al. [27]<br><br>Australia	14y (1,613)	FFQ (semi-quantitative)<br><br>(Past year)	212 individual foods, mixed dishes and beverages with standard serving sizes. CFC: never, rarely, number of times per month, per week and per day.	Parents and adolescents	Yes	3-day FD	(GL Ambrosini, HN de Klerk, TA O'Sullivan et al., unpublished results)<br><br>FFQ was able to correctly rank most nutrient intakes	25 Moderate risk of bias
Craig et al. [44]<br><br>Scotland	5-11y (721)<br><br>12-17y (512)	FFQ<br><br>(Last 2-3 mo)	Version C2 (5-11 y): 140 foods or drinks with a measure defined for each item.<br><br>Version C3 (12-13 y): Version C2 + six items covering intake of coffee and alcoholic drinks.	5-11 y (parent or guardian + child)<br><br>12-17 y (adolescent + parents or guardians)	Yes	4-day WFD	**Version C2** (Craig er al., 2010)<br>Spearman correlation coefficients: 0.21 to 0.56.<br>Significant (P<0.05) for all nutrients	25 Moderate risk of bias

							**Version C3** (Craig er al., 2010) Spearman correlation coefficients: 0.12 to 0.45. Significant for all nutrients except energy, total fat (% energy) and vitamins C and E..<br><br>The ranking agreement was better in younger children, absolute intakes agreed better between the two methods for older children.	
Bibiloni et al. [45]	12-17 y (1,231)	FFQ (semi-quantitative)	145 items (118 of the original	Adolescent	No	4-day FD	**Adults**	25

Spain		(Past year)<br><br>and 24h DR (2 non-consecutive days)	validated FFQ plus the most characteristic Balearic Islands foods) arranged by food type and meal pattern.<br>CFC: per day, week or month.				**Reproducibility** (Martin-Moreno et al., 1993) Pearson Correlation coefficients: 0.51 for saturated fat to 0.88 for alcohol.<br><br>**Validity** (Martin-Moreno et al., 1993) Pearson Correlation coefficients: 0.20 for vitamin A and 0.88 for alcohol	Moderate risk of bias
Aranceta et al. [64]<br><br>Spain	2-14y (3,534)	FFQ and 24h DR<br><br>24-h DR (1 day). A second 24-h DR was	164 items	Children under 8 y (child + mother or caregiver)	The authors stated that the FFQ was validated, but not cited the results	ND	ND	30<br>Low risk of bias

		applied in 25–30% of the sample						
Danyliw et al. [53]<br><br>Canada	2-18y (10,038)	24h DR<br><br>(1 day)	NA	≥12 y (adolescent)<br><br>6–11 y (children/ adolescent + parents or caregiver)<br><br>< 6 y (parents or caregiver)	NA	NA	NA	25 Moderate risk of bias
Smith et al. [20]<br><br>England	7y (6,056)	FFQ<br><br>(Nowadays)	94 food items. CFC: 5 responses ranging from: "never or rarely" to "more than once a day".	Mothers	No	Biochemical pa rameters	**In adults** (Rogers; Emmett, 1998) The FFQ produced mean nutrient intakes similar to those obtained for women in the National Diet and Nutritional Survey for British adults.	20 Moderate risk of bias

							The erythrocyte DHA content increased significantly with increasing frequency of consumption of oily fish (Rogers; Emmett, 1998)	
Northstone et al.[46]<br><br>England	13y (3,951)	FFQ<br><br>(Nowadays)	80 food items (the FFQ filled by the mothers)<br><br>54 food items (the FFQ filled by the adolescent)<br><br>CFC: 9 responses ranging from: "never or rarely" to "more than once a day".<br><br>The mother was asked specifically to respond to the	The mother filled the FFQ and the adolescent filled a short version of the FFQ	No	Biochemical pa rameters	As described by Smith et al. [20]	20 Moderate risk of bias

			questions only regarding the foods provided by her, including packed lunches but excluding school dinners and other foods consumed outside the home.<br><br>In the FFQ filled by the adolescent, they were asked about their consumption of foods that were not included in the mother's FFQ, (foods consumed as part of school dinners, food bought outside school and additional snacks and drinks).					

Northstone and Emmett [65]<br><br>England	4y (6,592)<br><br>7y (6,215)	FFQ<br><br>(Nowadays)	90 food items CFC: 5 responses ranging from: “never or rarely” to “ more than once a day”.	Mothers	No	Biochemical pa rameters	As described by Smith et al. [20]	20 Moderate risk of bias
Leventakou et al., [66]<br><br>Greece	4y (683)	FFQ<br><br>(Past year)	118 food items CFC: times per day, week, month and year or never.<br><br>The FFQ presented the following components: food frequency, type of meals during the day, use of dietary supplements, type of fat used for cooking, frequency of meals consumed in restaurants or	Primary caregivers	Yes	3 day FD	(Leventakou et al.,2014)<br><br>Weighted kappa statistics: 0·21 to 0·40 for most foods and nutrients.<br><br>The mean and median values of all food group and nutrient intakes did not differ significantly between the two dietary methods.	25 Moderate risk of bias

			take away and television viewing during meals). Parents could choose from one or two portion sizes. Seasonality of consumption was also reported in all food items.				On average, 88 % of participants were classified into the same or adjacent tertiles for nutrient and food group intakes by both dietary methods.	
Wall et al. [67]<br><br>New Zealand	3.5y<br>(550)<br><br>7y<br>(591)	FFQ<br><br>(Last mo)	71 food items<br>(3.5 years)<br><br>77 Food Items<br>(7 years).<br>CFC: 8 responses ranging from: never to 2 or more times per day.<br>Standard serving sizes were used as a reference for the core food group items.	Parents	No	4 day WFD and the biochemical status.	**In 6 to 24 months years**<br><br>**Reproducibility**<br>(Chua, 1999)<br>Spearman correlation coefficient: of the 54 foods, 44 of the foods had correlation of 0.5 or higher.	20<br>Moderate risk of bias

							**Validity** (Chua, 1999) Comparing food groups against nutrients resulted in fairly poor correlation except for read meat, breast milk and infant formula.	
Ovaskainen et al. [68] Finland	3y (708) 6y (841)	FD (3 non-consecutive days)	NA	Parents and day caregiver	NA	NA	NA	30 Low risk of bias
Durão et al. [69] Finland	4y (3,422)	FFQ (The previous six mo)	35 items. CFC: 9 responses ranging from Options: "never" to "≥4 times/day"	Child primary caregiver	Yes	3 days FD	(Durão et al., 2016) Pearson correlation coefficient: significant positive moderate were found for vegetable soup	25 Moderate risk of bias

							(r=0.54, P<0.001), fruit (r=0.42, P<0.001), milk (r=0.46, P<0.001) and yoghurts (r=0.48, P<0.001). ICC: 0.54 to 0.17	
Moreira et al.[49]<br><br>Portugal	5-10y (1,976)	FFQ (Last year)	86 food items. CFC: nine responses ranging from: never or less than once per month, to six or more times a day,	Parents	No	7-day FD as regards the fatty acid composition, with the composition of subcutaneous adipose tissue	**Adults** (Lopes et al., 2007)<br><br>Spearman correlation between FFQ and 7-day FD for fatty acid classes: 0.19 (trans isomers) to 0.72 (total saturated fat)<br><br>Spearman correlation between FFQ and adipose tissue for fatty acid classes: -	15 High risk of bias

							0.02 (trans isomers) to 0.44 (myristic)	
Borges et al. [47]<br><br>10 European cities	12.5-17.5y (2,213)	Computarized 24 h-DR (2 non-consecutive days)	ND	Adolescent	Yes	One day FD (study 1) and 24-h DR (study 2)	Study 1 (Vereecken et al., 2005) Spearman correlation: 0.44 to 0.79 Weighted Kappa: 0.11 to 0.55<br><br>Study 2 (Vereecken et al., 2005) Spearman correlation: 0.44 to 0.86 Weighted Kappa: 0.04 to 0.73	30 Low risk of bias

Manyanga et al. [70]<br><br>Australia<br>Canada<br>Finland<br>USA<br>Portugal<br>United Kington	9-11y<br>(3,274)	FFQ<br><br>(ND)	23 food groups.<br>CFC: 8 responses ranging from: never to more than once a day.	Children and adolescents	Yes<br>(for USA, Colombia and Finland)	3 days FD	**Reliability**<br>(Saloheimo et al., 2015)<br>ICC: 0.37 to 0.78.<br>Gross misclassification for all food groups was < 5%.<br><br>**Validity**<br>(Saloheimo et al., 2015)<br>Spearman correlation coefficients: below 0.5 for 22/23 food groups, and they differed among country sites<br>Gross misclassification was <5% for 22/23 food groups	20<br>Moderate risk of bias

Krusinska et al. [48]<br><br>Poland	13-18y (1,176)	FFQ (for Fruit/ Vegetable/ Fibre Intake<br><br>(Last year)	Nine dietary fibre sources.<br>CFC: less than once per week, once per week, 2–3 times per week, 4–6 times per week, every day	Adolescent	No	Multiple FD	**In adults**<br>Correlations coefficient: 0.65 for grams of fat and 0.40 for percentage energy from carbohydrate (Thompson and Byers,1994)	20<br>Moderate risk of bias
**Cross-sectional studies from Medium and Low Human Development Countries (MHDC and LHDC)**								
Araujo et al. [38]<br>Portugal	13y (1,489)	FFQ<br><br>(Last year)	91 food or beverage items.<br>CFC: nine responses ranging from: never to 6 times a day. It also included an open-ended section for foods not listed in the questionnaire, but eaten at least once a week.	Adolescents + parents or guardians	No	7-day FD as regards the fatty acid composition, with the composition of subcutaneous adipose tissue	**In adults**<br><br>As described by Moreira et al. 2010	20<br>Moderate risk of bias

Garba et al. [32]<br><br>Malaysia	13-17y (2,480)	FFQ<br><br>(Last mo)	126 items commonly eaten in Malaysia. CFC: 5 responses ranging from: never/rarely to daily intake. The serving size for each food item was also given according to the medium serving sizes in food serving size album and household measures were used for illustration	Adolescent	No	ND	ND	15 High risk of bias
Abdullah et al. [41]<br><br>Malaysia	12-19y (454)	FFQ<br><br>(Last year)	124 food items<br><br>CFC: ND	Adolescent + trained interviewers	Yes	3 days 24h DR	**Reproducibility** (Abdullah et al., 2012) Pearson correlation coefficient: 0.43 for carotene to 0.86 for total fat	25 Moderate risk of bias

							intake (median= 0.67)<br><br>**Validity** (Abdullah et al., 2012)<br>Pearson correlation coefficient: 0.22 (zinc) to 0.68 (calcium), median *r*-value of 0.43. Estimated mean intake for most nutrients assessed by the FFQ were higher as compared to the three DRs (p<0.05).<br><br>Most nutrients were classified into the same or adjacent	

							quartiles (median=52.7%).	
North et al. [22] England	3y (7,814)	FFQ (Nowadays)	CFC: 5 responses ranging from: never or rarely to more than once a day.	Mothers	No	Biochemical pa rameters	As described by Smith et al. [20]	20 Moderate risk of bias
Northstone and Emmett [25] England	2y (9,599)	FFQ (Nowadays)	53 items of foods and drinks. CFC: For milks and other drinks, this was recorded as times per week and for foods this was recorded as times per month.	Mothers	No	Biochemical pa rameters	As described by Smith et al. [20]	20 Moderate risk of bias
Silva et al. [31] Brazil	7-14y (1,136)	FFQ (Previous six mo)	132 food items. CFC: 4 responses ranging from: never to 5- 7 times a week	Children and Adolescents	Yes	2 day 24h DR	(Voci et al., 2011) Pearson correlation coefficient: -0.07 (iron) to 0.58 (vitamin C) Calibration coefficients:	20 Moderate risk of bias

							-0.07 (iron) to 0.40 (vitamin C)	
Nobre et al. [30]<br><br>Brazil	5y (232)	FFQ<br><br>(ND)	65 food items.<br>CFC: 5 responses ranging from: rarely or never to every day	Parents	No	ND	ND	15<br>High risk of bias
Pinho et al. [33]<br><br>Brazil	11-17y (535)	FFQ<br><br>(Previous six mo)	94 items.<br>CFC: 7 responses ranging from: never to 2 or more times a day.	Adolescent	Yes	2 days 24h DR	As described by Silva et al. [31]	25<br>Moderate risk of bias
Villa et al. [52]<br><br>Brazil	8- 9y (328)	FD<br><br>(3 non-consecutive days)	NA	Children + parents/ guardians	NA	NA	NA	30<br>Low risk of bias

Borges et al. [47] Brasil	12.5-17.5y (3,194)	FD (2 non-consecutive days)	NA	Adolescent (when the individual was unable to fill in the FD, this was completed with the help of another household member or a person that was appointed by the individual).	NA	NA	NA	30 Low risk of bias
Mais et al. [71] Brazil	2- 9y (929)	FFQ (7 days before the interview)	19 categories of foods based on their association with obesity, their high intake frequency in the Brazilian population, and recommendations of the Dietary Guidelines for the Brazilian Population.	Parents	No	ND	ND	10 High risk of bias

			CFC: 5 responses ranging from: not consumed to every day.					
Kehoe et al. [50] India	9.5y (538)	FFQ (Last mo)	136 items. CFC: daily, weekly or monthly.	Child + parent or guardian	No	ND	ND	15 High risk of bias
Nasreddine et al. [72] Lebanon	2-5y (525)	24h DR (1 day)	NA	Parents or caretaker	NA	NA	NA	25 Moderate risk of bias
Shang et al.[51] China	6-13y (5,267)	24h DR (3 consecutive days)	NA	Children and adolescents	NA	NA	NA	25 Moderate risk of bias
Wu et al. [60] China	5y (18,046)	FFQ (ND)	Eleven food groups. CFC: 5 responses ranging from: never to every day.	Parents	ND	ND	ND	15 High risk of bias

Manyanga et al., [70]<br><br>Brazil<br>Colombia<br>China<br>South Africa<br>India<br>Kenya	9-11y<br>(3,534)	FFQ<br><br>(ND)	As described by Manyanga et al. (2017) for HHDC	Children and adolescent	Yes<br>(for Colombia)	3 day FD	As described by Manyanga et al. [70] for HHDC	20<br>Moderate risk of bias

Abreviations: 24 H-DR - 24-hour dietary recall; CFC - Consumption frequencies categories; Comp. 24 h-DR – Computerized 24 hour dietary recall; FD - food diary; FFQ – food frequency questionnaire; ICC - intraclass coefficient correlation; Mo - months; NA – not applicable; ND – not described; UFD - unweighted food diary; WFD- weighed food diary.

References of the Reproducibility and validity studies

Chua, S. W. Y. *Iron and vitamin A nutrition of young Auckland children: an investigation into the methods to assess the nutritional status of micro-nutrients in 6-24 month olds: a thesis presented in partial fulfilment of the requirements for the degree of Master of Science in Nutritional Science at Massey University* (Doctoral dissertation, Massey University) **1999**

Chung, J.; Kwon, S. O.; Ahn, H.; Hwang, H.; Hong, S. J.; Oh, S. Y. Association between dietary patterns and atopic dermatitis in relation to GSTM1 and GSTT1 polymorphisms in young children. *Nutrients* **2015**, *7*(11), 9440-9452. DOI: 10.3390/nu7115473.

Craig, L. C. A.; McNeill, G.; Masson, L. F.; Macdiarmid, J.; Holmes, B.; Nelson, M.; Sheehy, C. Relative validity of two food-frequency questionnaires for children compared with 4-day diet diaries. *Proc Nutr Soc* **2010**, *69*(OCE6). DOI: 10.1017/S0029665110002910

Deschamps, V.; De Lauzon-Guillain, B.; Lafay, L.; Borys, J. M.; Charles, M. A.; Romon, M. Reproducibility and relative validity of a food-frequency questionnaire among French adults and adolescents. *Eur J Clin Nutr* **2009**, *63*(2), 282-291. DOI: 10.1038/sj.ejcn.1602914

Durão, C.; Severo, M.; Oliveira, A.; Moreira, P.; Guerra, A.; Barros, H.; Lopes, C. Association of maternal characteristics and behaviours with 4-year-old children's dietary patterns.
Matern Child Nutr **2016,** *Apr 3,* (Epub ahead of print) DOI 10.1111/mcn.12278.

Huybrechts, I.; Börnhorst, C.; Pala, V.; Moreno, L. A.; Barba, G.; Lissner, L.; et al. Evaluation of the Children's Eating Habits Questionnaire used in the IDEFICS study by relating urinary calcium and potassium to milk consumption frequencies among European children. *Int J Obes* **2011**, *35*(S1), S69-S78. DOI: 10.1038/ijo.2011.37.

Ireland, P.; Jolley, D.; Giles, G.; O'Dea, K.; Powles, J.; Rutishauser, I.; et al. Development of the Melbourne FFQ: a food frequency questionnaire for use in an Australian prospective study involving an ethnically diverse cohort. *Asia Pac J Clin Nutr* **1994**, *3*(1), 19-31.

Lanfer, A.; Hebestreit, A.; Ahrens, W.; Krogh, V.; Sieri, S.; Lissner, L.; et al. Reproducibility of food consumption frequencies derived from the Children's Eating Habits Questionnaire used in the IDEFICS study. *Int J Obes* **2011**, *35*(S1), S61-S68. DOI: 10.1038/ijo.2011.3.

Leventakou, V.; Georgiou, V.; Chatzi, L.; Sarri, K. Relative validity of an FFQ for pre-school children in the mother–child 'Rhea'birth cohort in Crete, Greece. *Public Health Nutr* **2015**, *18*(3), 421-427. DOI: 10.1017/S1368980014000445.

Lopes, C.; Aro, A.; Azevedo, A.; Ramos, E.; Barros, H. Intake and adipose tissue composition of fatty acids and risk of myocardial infarction in a male Portuguese community sample. *J Am Diet Assoc* **2007**, *107*(2), 276-286. DOI: 10.1016/j.jada.2006.11.008.

Martin-Moreno, J. M.; Boyle, P.; Gorgojo, L.; Maisonneuve, P.; Fernandez-rodriguez, J. C.; Salvini, S.; Willett, W. C. Development and validation of a food frequency questionnaire in Spain. *International journal of epidemiology* **1993**, *22*, 512-519.

Nurul-Fadhilah, A.; Teo, P.S.;Foo, L.H. Validity and reproducibility of a food frequency questionnaire (FFQ) for dietary assessment in Malay adolescents in Malaysia. *Asia Pac J Clin Nutr.* **2012**, 21 (1):97-103.

Rogers, I.; Emmett, P. Diet during pregnancy in a population of pregnant women in South West England. *Eur J Clin Nutr* **1998**, *52*, 246-250.

Saloheimo, T.; González, S. A.; Erkkola, M.; Milauskas, D. M.; Meisel, J. D.; Champagne, C. M., et al. The reliability and validity of a short food frequency questionnaire among 9–11-year olds: a multinational study on three middle-income and high-income countries. *Int J Obes Suppl* **2015**, *5*(S2), S22. DOI: 10.1038/ijosup.2015.

Thompson, F. E.; Byers, T. Dietary assessment resource manual. *J Nutr* **1994**, *124*(11), 2245S.

Vereecken, C. A.; Covents, M.; Matthys, C.; Maes, L. Young adolescents' nutrition assessment on computer (YANA-C). *Eur J Clin Nutr* **2005**, *59*, 658-667. DOI: 10.1038/sj.ejcn.1602124.

Voci, S. M.; Slater, B.; Silva, M. V. D.; Marchioni, D. M. L.; Latorre, M. D. R. D. D. Calibration study of the food frequency questionnaire for adolescents (AFFQ). *Cien Saude Colet* **2011**, *16*(4), 2335-2343.

Table S4. Risk of bias assessed by Meta Analysis of Statistics Assessment and Review Instrument (MAStARI) critical appraisal tools. Risk of bias was categorized as **High** when the study reaches up to 49% score "yes", **Moderate** when the study reached 50% to 69% score "yes", and **Low** when the study reached more than 70% score "yes".

2A- Cohort studies.

Question	Answer						
	Ambrosini et al.,2014	Fernandez-Alvira et al., 2015	Gatica et al., 2012	Lee et al.,2017	Lioret et al.,2015	Northstone et al.,2013	Camara et al. 2016
1. Is sample representative of patients in the population as a whole?	Y	N	Y	N	N	Y	N
2. Are the patients at a similar point in the course of their condition/illness?	Y	Y	Y	Y	Y	Y	Y
3.Has bias been minimized in relation to selection of cases and of controls?	NA	NA	NA	NA	NA	NA	NA
4. Are confounding factors identified and strategies to deal with them stated?	Y	Y	Y	Y	Y	Y	Y
5. Are outcomes assessed using objective criteria?	Y	Y	N	U	N	N	N
6. Was follow-up carried out over a sufficient time period?	Y	Y	Y	Y	Y	Y	Y
7. Were the outcomes of people who withdrew described and included in the analysis?	Y	Y	N	Y	Y	N	Y
8. Were outcomes measured in a reliable way?	N	U	N	N	N	N	N
9. Was appropriate statistical analysis used?	Y	Y	Y	Y	Y	Y	Y
% yes/risk**	77.7 L	66.6 M	55.5 M	55.5 M	55.5 M	55.5 M	55.5 M

*Y=Yes, N=No, U=Unclear, NA=Not applicable. **L=low risk, M=moderate risk, H=high risk

2B-Cross-sectional studies or longitudinal studies with cross-sectional analysis

Question		Answer												
	Abdullah et al., 2016	Ambrosini et al., 2009	Aranceta et al.,2003	Araujo et al. 2015	Bibiloni et al.,2011	Borges et al., 2018	Craig et al., 2010	Danyliw et al.,2011	Durão et al., 2016	Garba et al., 2014	Grieger et al.,2011	Kehoe et al., 2014	Krusinska et al., 2017	Leventakou et al.,2015
1) Was the study based on a	N	Y	Y	Y	Y	Y	N	U	Y	Y	Y	N	N	Y

random or pseudo random sample?														
2) Were the criteria for inclusion in the sample clearly defined?	Y	N	N	N	N	Y	N	Y	U	N	N	Y	Y	Y
3) Were confounding factors identified and strategies to deal with them stated?	Y	Y	U	Y	Y	Y	Y	N	Y	N	Y	Y	Y	Y
4) Were outcomes assessed using objective criteria?	Y	N	Y	N	N	Y	Y	N	Y	N	Y	N	N	Y
5) If comparisons are being made, was there sufficient description of the groups?	N	Y	N	Y	Y	N	N	Y	N	N	N	Y	N	Y
6) Was the follow up carried out over a sufficient time period?	Y	Y	Y	Y	Y	Y	Y	Y	Y	Y	Y	Y	Y	Y
7) Were the outcomes of people who withdrew described and included in the analysis?	N	Y	NA	NA	NA	NA	NA	NA	N	NA	NA	N	NA	N
8) Were the outcomes	Y	N	Y	N	Y	Y	Y	Y	N	N	Y	N	N	Y

measured in a reliable way?														
9) Was an appropriate statistical analysis used?	Y	N	U	Y	Y	Y	Y	Y	Y	N	Y	Y	Y	Y
% yes/risk	66.6 M	55.5 M	44.4H	55.5 M	66.6M	77.7 L	55.5M	55.5 M	55.5 M	22.2H	66.6 M	55.5 M	44.4 H	88.8 L

*Y=Yes, N=No, U=Unclear, NA=Not applicable. **L=low risk, M=moderate risk, H=high risk

2B-Cross-sectional studies or longitudinal studies with cross-sectional analysis

Question	Answer												
	Mais et al., 2017	Manyanga et al., 2017	McNaughton et al. 2008	Moreira et al.,2010	Nasreddine et al., 2017	Nobre et al., 2012	North et al., 2000	Northstone et al.,2014	Northstone e Emmett, 2013	Northstone et al. 2005	Oellingrath et al., 2010	Ovaskainen et al.,2009	Pinho et al., 2014
Was the study based on a random or pseudo random sample?	U	U	Y	Y	Y	Y	Y	Y	Y	Y	Y	N	Y
Were the criteria for inclusion in the sample clearly defined?	Y	U	N	N	Y	Y	Y	Y	Y	Y	N	Y	Y
Were confounding factors identified and strategies to deal with them stated?	Y	Y	N	Y	Y	Y	Y	Y	Y	Y	N	Y	Y
Were outcomes assessed using objective criteria?	N	N	N	N	N	N	N	Y	N	N	N	Y	Y
If comparisons are	Y	Y	Y	Y	Y	Y	Y	Y	Y	Y	Y	Y	Y

being made, was there sufficient description of the groups?													
Was the follow up carried out over a sufficient time period?	Y	Y	Y	Y	Y	Y	Y	Y	Y	Y	Y	Y	Y
Were the outcomes of people who withdrew described and included in the analysis?	NA	NA	NA	NA	NA	N	N	Y	Y	N	NA	N	NA
Were the outcomes measured in a reliable way?	N	U	N	N	Y	N	N	Y	N	N	N	Y	N
Was an appropriate statistical analysis used?	Y	Y	Y	Y	Y	Y	Y	Y	Y	Y	Y	Y	Y
% yes/risk	55.5<br>M	44.4<br>H	44.4<br>H	55.5<br>M	77.7<br>L	66.6<br>M	66.6<br>M	100<br>L	77.7<br>L	66.6<br>M	44.4<br>H	77.7<br>L	77.7<br>L

*Y=Yes, N=No, U=Unclear, NA=Not applicable. **L=low risk, M=moderate risk, H=high risk

2B-Cross-sectional studies or longitudinal studies with cross-sectional analysis

Question	Shang et al., 2012	Silva et al., 2012	Smith et al., 2011	Villa et al., 2015	Wall et al., 2013	Wu et al., 2017
Was the study based on a random or pseudo random sample?	Y	N	Y	Y	N	Y
Were the criteria for inclusion in the sample clearly defined?	N	N	Y	Y	Y	Y
Were confounding factors identified and strategies to deal with them stated?	N	Y	Y	Y	Y	Y
Were outcomes assessed using objective criteria?	Y	N	N	Y	N	N

If comparisons are being made, was there sufficient description of the groups?	Y	Y	Y	Y	N	Y
Was the follow up carried out over a sufficient time period?	Y	Y	Y	Y	Y	Y
Were the outcomes of people who withdrew described and included in the analysis?	NA	NA	Y	NA	N	N
Were the outcomes measured in a reliable way?	Y	U	N	Y	N	N
Was an appropriate statistical analysis used?	N	Y	Y	Y	Y	Y
% yes/risk	55.5<br>M	44.4<br>H	77.7<br>L	88.8<br>L	44.4<br>H	66.6<br>M

*Y=Yes, N=No, U=Unclear, NA=Not applicable. **L=low risk, M=moderate risk, H=high risk
